# Distinct functional responses of root endophyte and rhizosphere microbial communities in intercropping systems under arid conditions

**DOI:** 10.3389/fpls.2026.1809801

**Published:** 2026-06-30

**Authors:** Sulamita Santos Correa, Aysha Hamad Mohammed Asayan Almansoori, Shahnaz Nazzar, Balamurugan Sadaiappan, Qurban Ali, Ponnarmadha Subramani, Sunil Mundra

**Affiliations:** 1 Department of Biology, College of Science, United Arab Emirates University, Al Ain, United Arab Emirates; 2 School of Biology, Indian Institute of Science Education and Research, Thiruvananthapuram, India; 3 Khalifa Center for Genetic Engineering and Biotechnology, United Arab Emirates University, Al Ain, United Arab Emirates

**Keywords:** agroecosystems, co-cultivation, dryland, plant growth-promoting bacteria (PGPB), soil microbiome

## Abstract

**Introduction:**

Sustainable strategies have been implemented to enhance plant development and productivity, including intercropping systems. This approach is particularly effective in arid regions, where diverse microbial populations associated with intercropping plants contribute significantly to stress tolerance and plant growthpromoting.

**Methods:**

We evaluated how intercropping and monocropping systems influence the diversity, functional traits, and stress tolerance of root-associated bacteria. Root endophytic and rhizosphere bacteria were isolated from intercropping and monocropping systems under arid conditions and evaluated for plant growth-promoting traits, enzymatic activities, exopolysaccharide and cellulose production, biofilm formation under drought stress, and tolerance to drought, salinity, and heat stress.

**Results and discussion:**

Eighty bacterial isolates were characterized, most of which exhibited multiple plant growth-promoting traits and tolerance to environmental stress. Overall, *Bacillus* spp. were the dominant bacteria among endophyte and rhizosphere communities. In alfalfa-broad and Egyptian wheat-broad intercropping, *Bacillus* spp. were more common, whereas *Pseudomonas* spp. were more common in barley-mustard intercropping. Multivariate analysis of functional traits (NMDS) revealed that bacterial communities were primarily structured by niche (endophytic vs. rhizosphere) rather than by intercropping type. The results suggest that rhizosphere bacteria associated with intercropping enhanced nitrogen fixation compared to those from monocropping systems, and the exopolysaccharide produced by endophytic isolates from intercropping using glucose as a carbon source varied from monocropping. Based on our results, the intercropping system creates a favorable microenvironment for certain bacteria, such as *Bacillus* spp. and *Pseudomonas* spp., which possess specific plant growth-promoting traits suitable for harsh environments, such as arid regions. These findings support other studies showing that bacteria adapted to extreme conditions, and isolated from diverse and multiple cropping systems, can function as plant bioinoculants, supporting plant species under adverse conditions.

## 1 Introduction

Climate change represents significant challenges for land use, particularly in arid and drought regions where soil degradation and reduced fertility threaten crop productivity. To address these challenges, appropriate agricultural practices such as intercropping are required to maintain stable yields and soil functionality (Wang et al., 2021; Ameer et al., 2023). However, although intercropping systems provide several agronomic and ecological benefits (Benard et al., 2023), their performance may vary depending on crop combinations, environmental conditions, and soil microbial interactions. Competition between crops for water, nutrients, and light can negatively affect productivity, particularly under arid and nutrient-poor conditions (Fares and Mamine, 2023). This limitation restricts the development of efficient microbial-based strategies to improve crop productivity and stress tolerance under harsh environmental conditions.

In this context, plant growth-promoting bacteria (PGPB) have gained considerable attention in agriculture as biofertilizers due to their ability to enhance plant growth through multiple mechanisms, including nutrient acquisition, phytohormone production, and stress mitigation (Ali et al., 2025; Zhang et al., 2023; Zheng et al., 2024). Understanding these complex interactions between intercropping plants and their associated microbial communities is crucial, as the mutualistic cooperation between plant hosts and microbes is largely driven by genetic diversity and biochemical approaches (Lee et al., 2024; Sadaiappan et al., 2025). Such interactions help explain the stability of variations in mutualistic traits observed in nature. The selection and evaluation of crop plant-microbiome associations should also be guided by qualitative, trait-based, and quantitative analyses of microbiome-associated plant phenotypes (Oyserman et al., 2021; He et al., 2023). Moreover, for genetic improvement to be achieved, a solid understanding of the physiological and biochemical changes in plants induced by PGPB is required.

Despite these advances, most previous studies have focused primarily on microbiome diversity, taxonomic and gene composition, with limited attention to the functional capabilities and stress tolerance of bacterial isolates (Ali et al., 2025; Khan et al., 2023). This emphasis on culture-independent approaches is partly associated with the technical and labor-intensive nature of isolating, screening, and characterizing large numbers of cultivable bacteria for plant growth-promoting (PGP) traits and abiotic stress tolerance (Jiménez et al., 2026). Consequently, the extent to which functional traits differ between endophytic and rhizosphere communities under cropping systems remains less explored (Xun et al., 2024). This distinction is especially relevant in arid environments, where microbial adaptations to drought, salinity, and heat are critical for plant survival and productivity (Metze et al., 2023; Fazal et al., 2025). Therefore, understanding how these microbial niches differ functionally under intercropping and monocropping conditions may provide insights into plant-microbe interactions and contribute to improved crop performance under stress conditions.

In this study, we aimed to isolate, identify, and functionally characterize the bacterial community from the roots and rhizosphere of different intercropping and monocropping systems. We assessed the mechanisms employed by these bacterial isolates for PGP traits and abiotic stress tolerance. Additionally, we conducted a comparative analysis of the physiological traits and metabolic profiles of these rhizosphere and endophytic bacteria within their host plant to better understand their roles in promoting plant health and productivity. This collection of microbes could be used in future experiments to test the effects of these endophytes and rhizosphere isolates on plant growth and stress tolerance promotion to improve agricultural crop production in arid and hot regions.

## 2 Material and methods

### 2.1 Site description and experimental design

**Figure 1:**
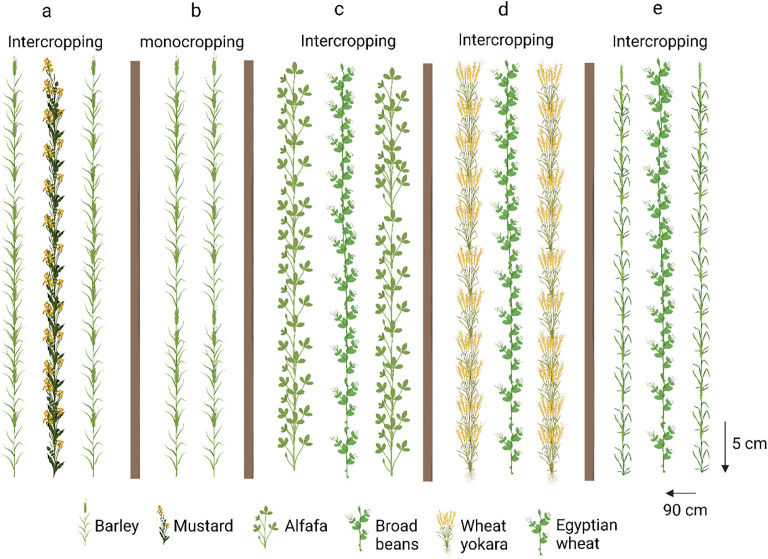
Schematic diagrams of the field experiment. **(a)** Barley/Mustard intercropping; **(b)** Barley monocropping; **(c)** Alfalfa/Broad beans intercropping; **(d)** Wheat yokara/Broad beans intercropping; **(e)** Egyptian wheat/Broad beans intercropping. Each treatment included 25 replicate plants. (Created with BioRender.com).

A field experiment was conducted by from October 2023 to March 2024 at Al Foah Farm, (KCGEB, UAE University), located in Al Ain, United Arab Emirates (UAE) (24.356806, 55.799162). The site is characterized by sandy soil and winter temperatures ranging from 20 °C to 26 °C. During the crop growing season (October 2023–March 2024), the mean air temperature was 23.5 °C, with an average range of 14.8 °C to 34.9 °C, as accessed from the NASA POWER Data Viewer (https://power.larc.nasa.gov/data-access-viewer/). The total precipitation during the study period was 173.8 mm (**Supplementary Table 1**). A completely randomized field plot design with five treatments (25 replicates per treatment) comparing monocropping and intercropping system was conducted: A1: barley (*Hordeum vulgare*)/mustard (*Brassica juncea*); A2: barley (monocropping); A3: alfalfa (*Medicago sativa*)/broad beans (*Vicia faba*); A4: wheat yokara (*Triticum aestivum*)/broad beans; A5: Egyptian wheat (*Triticum turgidum*)/broad beans (**Figure 1**). Row and plant spacing were maintained at 90 cm and 5 cm, respectively. Four plant root and rhizosphere soil samples (approximately 0–15 cm around the plants) were randomly collected from each treatment group during the developmental stage (≈35–45 days after seed emergence). Roots were excavated intact, and rhizosphere soil was isolated by removing loosely adhering particles. Samples were stored at 4 °C until analysis.

### 2.2 Endophytic and rhizosphere bacterial isolation

For endophytic bacteria, roots were surface-sterilized using 70% ethanol (10s) and 2.5% sodium hypochlorite (NaClO) for 3 min, followed by multiple rinses with distilled water. Sterilized tissues were macerated, and 10 g of the resulting material was suspended in 100 mL of sterile saline solution and filtered through a sterile filter. Serial dilutions (10^-2^ to 10^-6^) were plated (0.2 mL) onto Luria Bertani (LB) agar (medium composition provided in **Supplementary Table 2**) supplemented with 50 µg/mL of nystatin and cycloheximide (Sigma-Aldrich Chemie GmbH, Germany) to suppress fungal growth. After incubation at 30 °C, colonies were purified by repeated streaking on Nutrient Agar (NA). Rhizosphere soil (1–3 mm layer) was collected, and 10 g was suspended in 90 mL of sterile saline water. To dislodge bacterial cells, the suspension was sonicated at 150 W for 30 s. Serial dilutions (10^-2^ to 10^-6^) were plated onto LB agar (50 µg/mL cycloheximide) and incubated at 30 °C for 48 h. Representative colonies were purified on NA medium at 30 °C for 24 h.

### 2.3 DNA extraction, 16S rRNA sequencing

DNA was extracted from isolates cultured in nutrient broth (NB) medium at 100 rpm for 24 h at 30 °C, using the DNeasy Power Soil kit (Qiagen) following the manufacturer’s instructions with some modifications. Previously, in the DNA kit steps, cells were lysed (480 µL of EDTA 0.5 M and 120 µL of lysozyme 100 mg/mL) using mechanical disruption. Total DNA was quantified using a NanoDrop spectrophotometer (Thermo Fisher Scientific), and DNA integrity was evaluated by non-denaturing agarose gel electrophoresis (1.5%) for 60 min at 90 volts. Subsequently, 16S rDNA was amplified by PCR using the bacterial universal primers 27f and 1492r (Lane, 1991). PCR reaction was performed in a total volume of 20 μL, containing 1 μL of each primer, 2 μL of AmpliTaq Gold 360 Master Mix, 15 μL of water, and 1 μL of bacterial genomic DNA. PCR cycling conditions were: 95 °C for 5 min, 30 cycles of 95 °C for 30 s, 50 °C for 40 s, 72 °C for 2 min, and a final extension at 72 °C for 7 min. Amplicons were evaluated by non-denaturing agarose gel electrophoresis (1.5%) for 60 min at 90 volts and sequenced by the Sanger method. Sequences were processed in BioEdit, and taxonomy identification of isolates was determined using NCBI BLAST based on the closest reliable match, considering sequence similarity and query coverage (sequences showing ≥98% similarity were considered for species-level). Phylogenetic analysis was performed in MEGA (v11) using the Maximum Likelihood and Neighbor-Joining method (Tamura et al., 2021). The final phylogenetic tree with enzyme annotation was created using EvolView V3.

### 2.4 *In vitro* screening for nitrogen fixation ability

The isolates were evaluated to fix atmospheric nitrogen (N) by growing on Jensen’s agar, an N-free medium, in triplicate at 30 °C for 24 h. Colony formation on this medium was considered indicative of potential N-fixing capacity. Ammonia production as a product of N fixation (Shomi et al., 2021) was measured in peptone water at 33 °C, shaking at 150 rpm for 24 h. Cultures were centrifuged at 4,000 g for 20 min, and ammonia in the supernatant was determined using Nessler’s reagent (light-yellow indicated low production and dark yellow to brown indicated higher production), measuring at 450 nm (spectrophotometer Promega, GloMax Discover) and comparing it with a standard curve prepared using ammonium sulfate (0.1-1 µmol/mL).

### 2.5 Indole-3-acetic acid, exopolysaccharide, cellulose production, and biofilm formation

IAA (indole-3-acetic acid) production was evaluated following the method according to Correa et al. (2024), with slight modifications. Isolates were cultured in NB with 150 μg/mL of L-tryptophan and incubated at 30 °C, shaking at 150 rpm for 3 d. Culture was then centrifuged at 5,000 rpm for 10 min. Following, 150 μL of the supernatant was mixed with 100 μL of Salkowski’s reagent in a 96-well plate and incubated in the dark for 30 min with gentle shaking. IAA production was measured at 540 nm (spectrophotometer Promega, GloMax Discover). IAA concentrations were estimated from a standard curve prepared with synthetic IAA (25 to 600 μg/mL).

Exopolysaccharides (EPS) production was evaluated using Yeast Mannitol (YM) medium (Ventorino et al., 2019). In this assay, mannitol was replaced with either sucrose or glucose (10%, w/v). Cultures were incubated in triplicate at 30 °C under shaking at 150 rpm for 3 d. Aliquots of 30 μL were then plated onto YM agar containing the carbon sources and incubated at 30 °C for 48 h. Formation of mucoid colonies and ethanol precipitation indicated a positive result. Carbohydrate content of the EPS was determined using the phenol-sulfuric acid method as described by DuBois et al. (1956), with minor modifications. Cultures were centrifuged at 10,000 × g for 20 min at 4 °C, and the supernatant was stored at -20 °C for 4 d to allow EPS precipitation. The precipitates were resuspended after centrifugation in 5% phenol solution and 2.5 mL of sulfuric acid and mixed at room temperature for 20 min, followed by incubation. The absorbance was measured at 490 nm, and the carbohydrate concentration was determined based on a standard curve ranging from 10 to 100 μg mL−1.

For cellulose production, the isolates were cultivated at 30 °C for 4 d in Glucose-Yeast Ethanol Extract Agar (GYEA) containing: 2% D-glucose, 1% yeast extract, 5% ethanol, 0.3% CaCO3, and 2% agar. After incubation, the clear zones around each isolate indicated their cellulose production efficiency. In addition, biofilm formation by isolates was assessed under drought stress and non-stress conditions using 10% PEG-8000 (-1.69 MPa osmotic potential) as described by Michel and Kaufmann (1973). The isolates were grown in 96-well plates containing NB with 0% or 10% of PEG-8000 and incubated at 30 °C for 24 h with shaking (120 rpm). After incubation, wells were washed five times with sterile ultrapure water to remove non-adherent cells and air-dried. Biofilms were stained with 1% crystal violet for 45 min, washed, and dried again. The bound dye was then solubilized with absolute ethanol, and biofilm formation was quantified by measuring absorbance at 560 nm using a spectrophotometer (Promega, GloMax Discover).

### 2.6 Assessment of siderophore production, phosphate, and potassium solubilization

Siderophore production was evaluated using NA supplemented with 10% Azurol S (CAS). The CAS reagent was prepared by combining 20 mL of CTAB (cetyltrimethylammonium bromide) 5 mM solution, 20 mL of Chrome Azurol 2.5 mM solution, and 5 mL of FeCl3–1 mM solution. To prevent iron contamination, all materials were pre-treated overnight with 10% HCl. Bacterial cultures in NB were spotted (10 μL) in triplicate onto CAS agar plates and incubated at 30 °C for 3 d. Siderophore production was indicated by the appearance of color change halos surrounding the colonies, resulting from iron chelation (Sun et al., 2022).

The potential of the isolates to solubilize phosphate was evaluated on Pikovskaya’s agar. Isolates were inoculated in triplicate at 30 °C for 48 h. Phosphate solubilization was confirmed by the formation of a clear halo around the colonies, demonstrating the release of phosphate from insoluble compounds (Sanchez-Gonzalez et al., 2022). Potassium solubilization was assessed using Aleksandrow agar. After inoculation (30 °C for 48 h), a translucent halo around the colonies indicated positive potassium solubilization (Raji and Thangavelu, 2021).

### 2.7 Screening for enzymatic activities

ACC (1-aminocyclopropane-1-carboxylic acid) deaminase activity was assessed using sterile DF (Dworkin and Foster) minimal medium supplemented with 3 mM ACC as the sole N source (Penrose and Glick, 2003). Isolates were pre-cultured in NB (30 °C, 120 rpm, 24 h), washed with 0.1 M Tris-HCl, and inoculated onto DF-ACC plates. Growth was monitored at 30 °C for 7 days, with ammonium sulfate serving as a positive control.

Extracellular enzymatic activities were determined by inoculating isolates onto NA medium (incubated at 28 °C for 4 d) supplemented with specific substrates (Ayaz et al., 2024). Amylase activity was determined using NA medium supplemented with 1% starch, and Lugol’s iodine solution (1%) was added to the plates. Clear halo zones around the colony indicated the presence of amylase activity. Cellulase activity was determined using the NA medium supplemented with carboxymethyl (CMC). The medium was covered with Congo red solution, and clear zones from pink to yellow around the bacterial colony indicated the presence of cellulase enzyme activity. Protease activity was determined using NA medium enriched with 1% skim milk, and the presence of transparent or white halo zones surrounding the colony indicated proteolytic activity. Lipase activity was evaluated using NA medium supplemented with 3% tributyrin as the substrate; clear halo zones surrounding the bacterial colonies indicated the hydrolysis of tributyrin, demonstrating lipase activity (Toghueo et al., 2017). Xylanase activity was evaluated using NA medium enriched with xylan; the plates were flooded with a 1% Congo red solution and then washed with 1 M NaCl to visualize zones of xylan degradation. A distinct, clear zone around the colonies indicated xylanase activity (Samanta et al., 2011).

### 2.8 Assessment of abiotic stress tolerance: drought, heat, and salinity

Drought stress was simulated by cultivating the isolates in triplicate (initial OD600 of 0.1) under an osmotic potential of -1.69 MPa, achieved by dissolving 100g/L (10%) PEG-8000 in NB medium or without PEG-8000 (control), at 30 °C, 120 rpm for 48 h. Following growth was estimated by measuring OD600, and the osmotic potential was calculated using an equation that relates to PEG-8000 concentration (Michel and Kaufmann, 1973; Sandhya et al., 2009):


Ψ = 1.29[PEG]2T − 140[PEG]2 − 4.0[PEG]

where Ψ is the water potential of each treatment (bars), [PEG] is the concentration of PEG solution [g PEG (g H2O) -1], and T is temperature (°C).

For heat, the isolates were inoculated into NB medium at an initial OD600 of 0.1, at 30 °C, 120 rpm, for 48 h, in different temperatures (40 °C, 45 °C, and 55 °C). For salinity stress, the isolates were inoculated into NB medium (supplemented with 5% or 10% NaCl) at an initial OD600 of 0.1 (30 °C, 120 rpm, for 48 h). The tolerance to heat and salinity was estimated by measuring OD600 in triplicate.

### 2.9 Statistical analysis

The PGP traits of endophyte and rhizosphere communities were compared between intercropping and monocropping systems. The data was analyzed using analysis of variance (ANOVA) from three replicates. Normality of the data was assessed using the Shapiro-Wilk test. The Student’s t-test was performed on the data with a normal distribution, whereas the non-parametric Mann-Whitney test was applied when the assumption of normality was not satisfied. Comparisons between independent groups were performed using independent two-sample t-tests (Welch t-test). Differences were considered statistically significant at P < 0.05. The frequency of cultured bacterial genus was calculated based on the total number of isolates recovered and expressed as the proportion of isolates assigned to each taxon. For investigating the functional patterns of root endophytic and rhizosphere soil bacteria communities, as well as communities of different intercropping systems, non-metric multidimensional scaling (NMDS) and PERMANOVA analyses were performed. Statistical analyses were performed using RStudio software, version 2024.09.0 + 375.

## 3 Results

### 3.1 Bacterial community composition and taxonomic diversity

**Figure 2:**
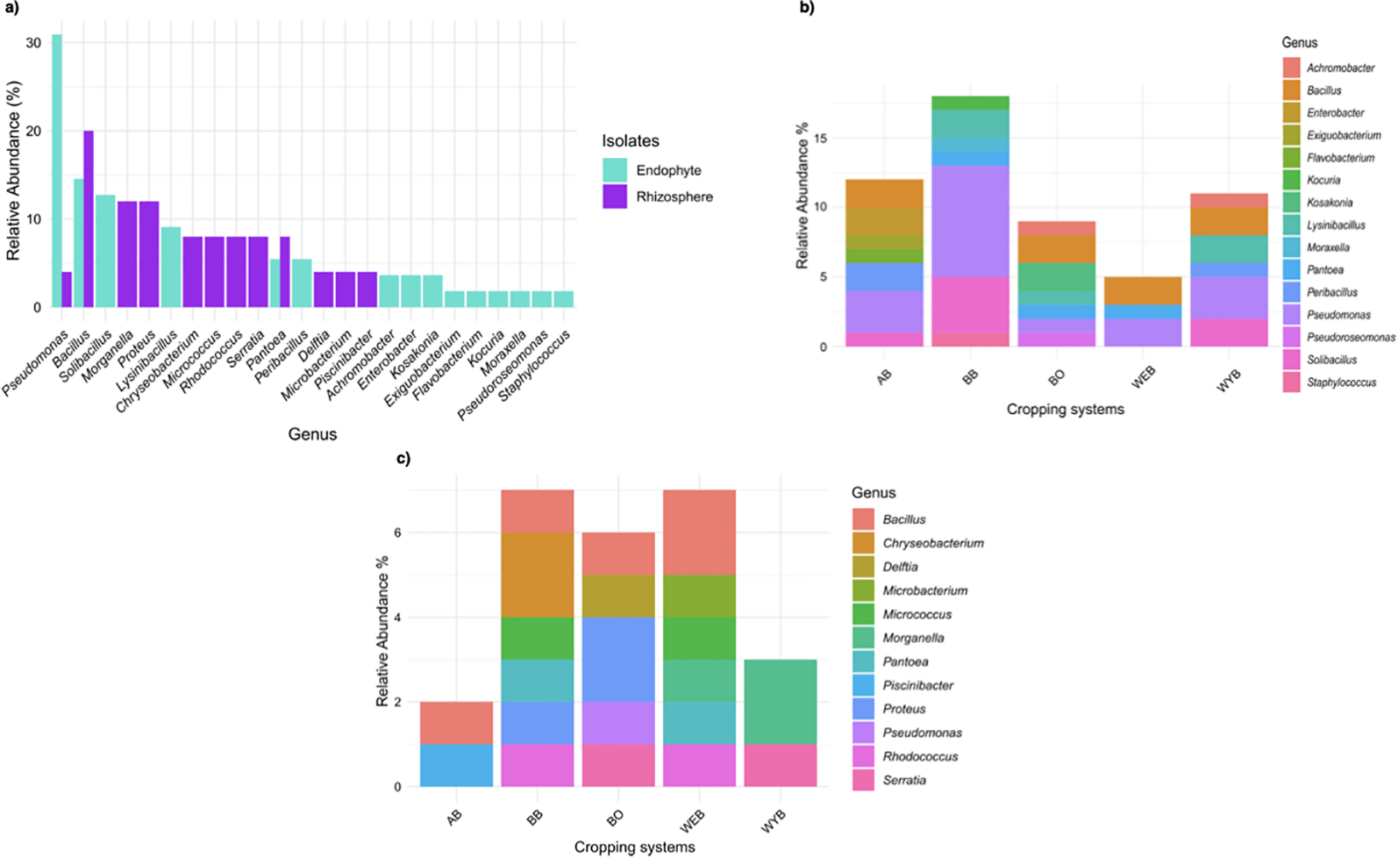
Frequency of bacterial genus among endophytic and rhizosphere isolates **(a)**. Frequency of bacterial genera of endophytic isolates **(b)** and rhizosphere isolates **(c)** across the different cropping systems. (AB) alfalfa/broad beans; (BB) barley/mustard; (BO) barley monocropping; (WEB) Egyptian wheat/broad beans; (WYB) wheat yokara/broad beans. The data are presented as relative abundance for descriptive comparison, and no inferential statistical test was applied. (Created with BioRender.com).

A total of 80 bacteria were isolated, comprising 55 root endophytes and 25 rhizosphere isolates. Taxonomic analysis revealed distinct distribution patterns between the two niches (**Figure 2a**). *Pseudomonas* (>30%) and *Bacillus* (>14%) dominated the endophytic community, whereas the rhizosphere was characterized by a prevalence of *Bacillus* (>18%), followed by *Morganella* and *Proteus* (>10%).

**Figure 3:**
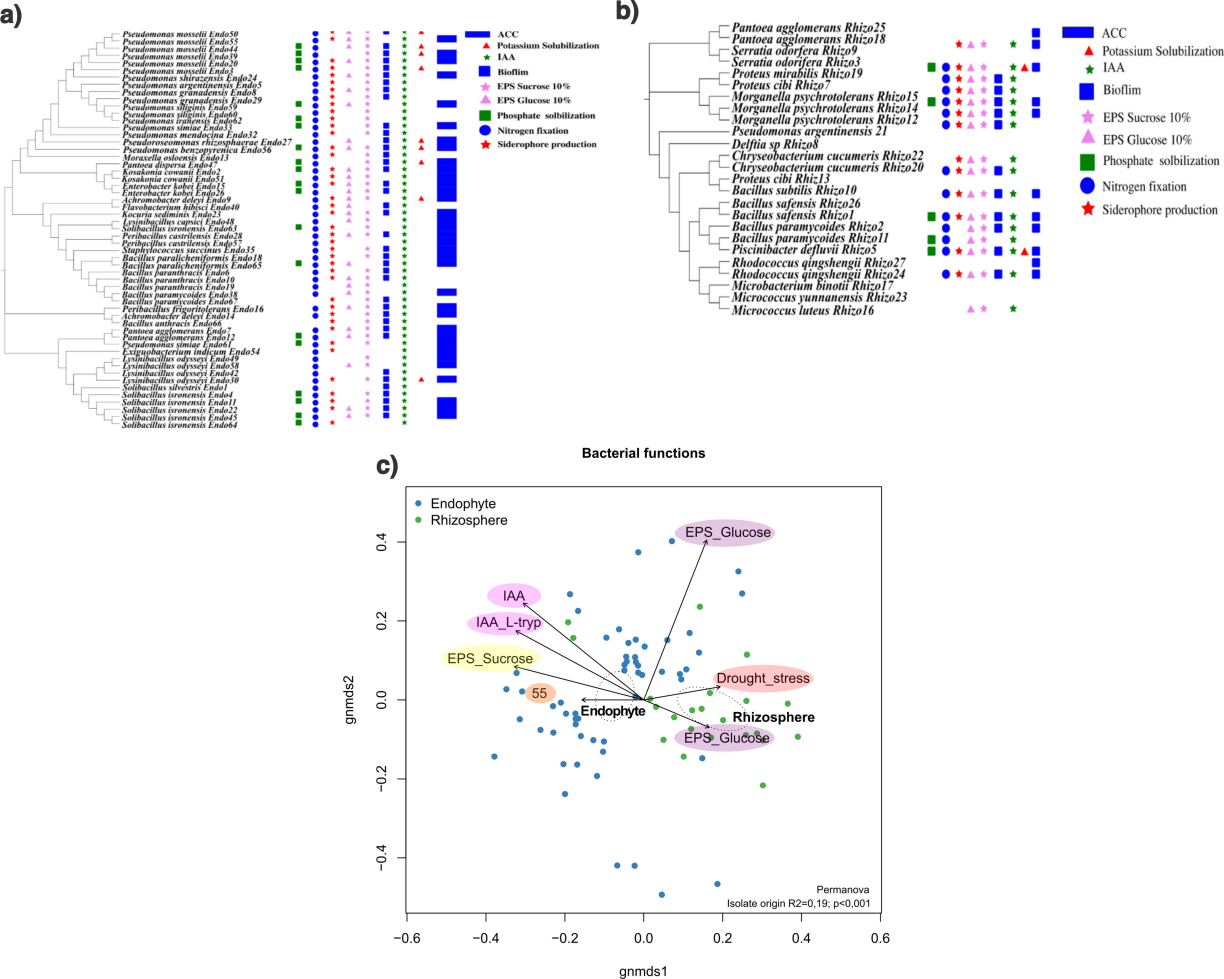
Phylogeny and functional diversity of bacterial communities. Phylogenetic trees for **(a)** endophytic and **(b)** rhizosphere isolates, showing relative abundance and enzymatic potential. **(c)** NMDS plot displaying functional trait distribution (nutrient cycling, phytohormone signaling, and tolerance to drought, heat, and salinity) across all cropping systems. Vectors represent significant environmental/functional drivers fitted using a permutation test (envfit, 99 permutations; p < 0.05). Dotted ellipses show 95% confidence intervals around group centroids based on 25 biological replicates. (Created with BioRender.com).

In all intercropping combinations (alfalfa-broad bean, barley-mustard, Egyptian wheat-broad bean, and wheat yokara-broad bean), the endophytic communities were consistently dominated by *Pseudomonas* (**Figure 2b**). A similar trend was observed in the rhizosphere of these systems, where *Bacillus* was the most prevalent genus, particularly in alfalfa-broad bean, barley-mustard, and Egyptian wheat-broad bean treatments (**Figure 2c**). In contrast, barley monocropping shifted the microbial structure, with *Kosakonia* dominating the endosphere and *Piscinibacter* prevailing in the rhizosphere (**Figures 2b, c**). High-confidence species-level identification confirmed that these isolates belong to diverse genera associated with plant growth-promoting (PGP) traits (**Figures 3a, b**).

### 3.2 Functional structure and source-dependent traits

NMDS ordination and PERMANOVA indicated that bacterial functional profiles were more strongly shaped by their isolation source (endophyte vs. rhizosphere) rather than the intercropping combination (**Figure 3c**; **Supplementary Table 3**). No significant interaction was found between the isolation source and the cropping system regarding functional structure. Marked functional differences were observed between niches: rhizosphere isolates showed superior drought tolerance and higher EPS production (with glucose), while endophytic isolates were characterized by higher IAA production (both with and without tryptophan), sucrose-dependent EPS production, and enhanced thermal tolerance at 55 °C.

### 3.3 Plant growth-promoting characteristics

**Figure 4:**
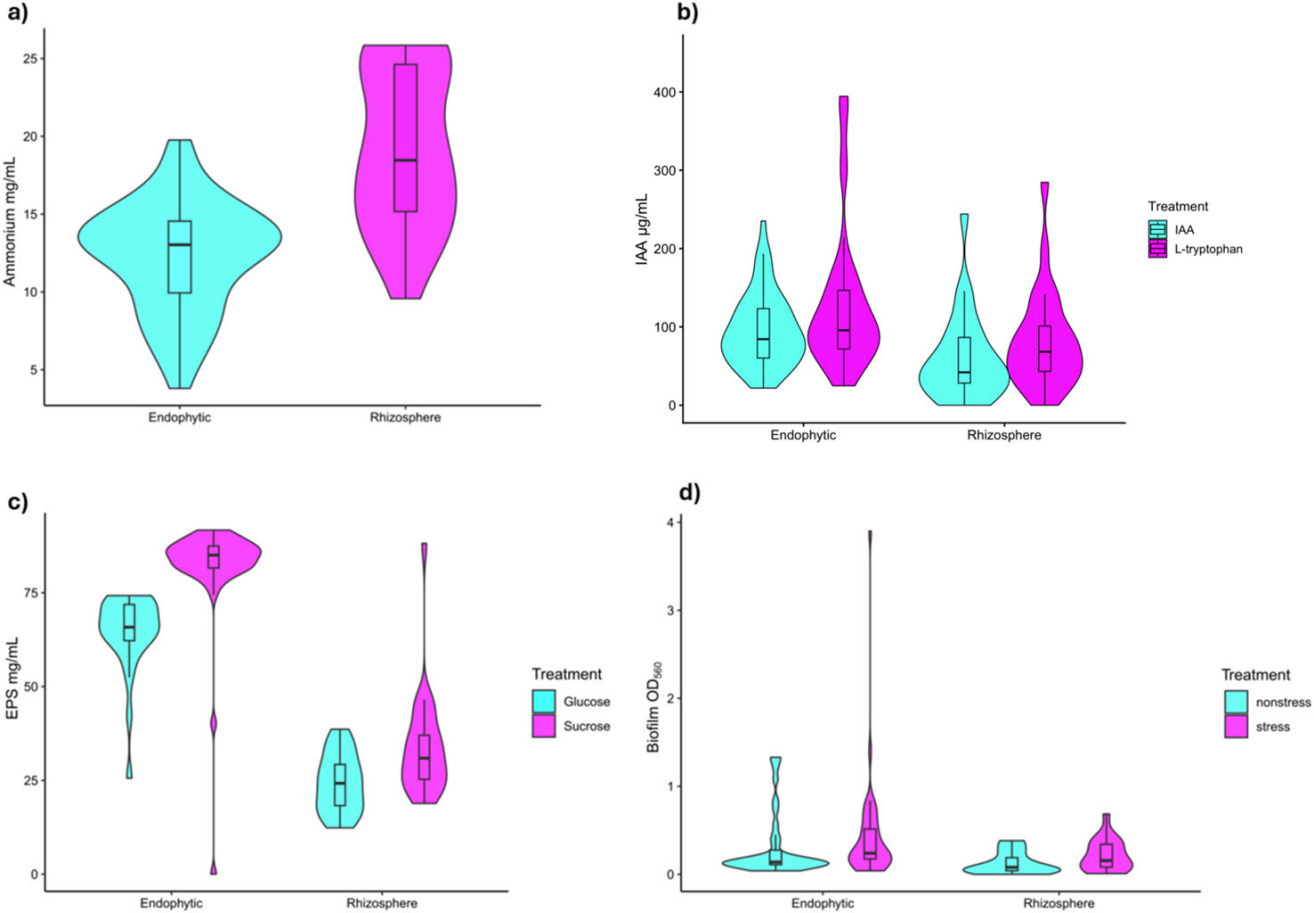
Quantitative estimation of PGP traits of root endophytic (turquoise) and rhizosphere (purple) isolates from intercropping and monocropping systems. **(a)** Ammonia production. **(b)** IAA production in the absence of 150 μg/mL L-tryptophan. **(c)** EPS production in media supplemented with 10% glucose or sucrose. **(d)** Biofilm formation under non-stress and drought stress conditions. Bars represent mean values ± standard deviation (n = 3). Data normality was evaluated using the Shapiro-Wilk test, and statistical differences between endophytic and rhizosphere isolates were determined using Welch’s two-sample t-test. Statistical significance was considered at *P < 0.05*. (Created with BioRender.com).

Biochemical characterization of the 80 isolates revealed a diverse range of functional PGP traits (**Figure 4**). All endophytic and rhizosphere strains indicated N-fixation capability. However, ammonia production was significantly higher in rhizosphere isolates compared to endophytic ones (19.06 vs. 12.28 µg/mL; t=-5.60, *p=0.001*) (**Figure 4a**). Among the top-performing ammonia producers, endophytic strains were represented by *Pseudoroseomonas rhizosphaerae* (Endo27), *Pseudomonas mosselii* (Endo39), and *Exiguobacterium indicum* (Endo54), among others (**Supplementary Table 4**). The most efficient rhizosphere producers included *Bacillus paramycoides* (Rhizo2), *Proteus mirabilis* (Rhizo19), and *Chryseobacterium cucumeris* (Rhizo22) (**Supplementary Table 5**).

IAA production was assessed with and without L-tryptophan supplementation. For both niches, the addition of L-tryptophan significantly enhanced IAA yields (*p<0.05*). Notably, endophytic bacteria produced significantly higher IAA concentrations than rhizosphere isolates under both conditions (**Supplementary Table 4**). In the absence of L-tryptophan, endophytes produced an average of 95.73 μg/mL compared to 59.62 μg/mL for rhizosphere isolates (*p=0.007*). With L-tryptophan supplementation, endophytic production increased to 128.76 μg/mL, remaining significantly higher than the rhizosphere average of 82.07 μg/mL (*p=0.011*).

Among endophytes, *Pseudomonas argentinensis* (Endo5) and *Enterobacter kobei* (Endo26) were the top producers in media with and without L-tryptophan, respectively (**Supplementary Table 4**). For rhizosphere isolates, the highest yields were observed in *Morganella psychrotolerans* (Rhizo12) and *Bacillus subtilis* (Rhizo10) (**Supplementary Table 4**). In addition, EPS production (expressed as mg/mL of glucose or sucrose equivalents in the culture supernatant, as determined by the phenol-sulfuric acid method) was significantly influenced by both the carbon source and the isolation niche (**Figure 4c**). In sucrose-containing medium, endophytic isolates produced significantly higher EPS levels than rhizosphere bacteria (71.37 vs. 33.91 mg/mL; p<0.001), whereas no significant difference was found in glucose-containing medium (*p=0.061*). Notably, both groups exhibited a marked preference for sucrose, with endophytes showing a more substantial increase in EPS yield compared to glucose (*p<0.001*). Among the endophytic isolates, 47 strains produced EPS in sucrose, led by *Pantoea agglomerans* (Endo31), while 28 strains were active in glucose, with *Enterobacter kobei* (Endo15) reaching the maximum yield (74.25 mg/mL) (**Supplementary Table 4**). All rhizosphere isolates were EPS-producers under both conditions, with *Bacillus subtilis* (Rhizo10) and *Serratia odorifera* (Rhizo9) showing the highest production in sucrose and glucose, respectively (**Supplementary Table 4**).

Biofilm formation, quantified by crystal violet staining (OD_560_), was compared under non-stress and drought-stress (-1.69 MPa) conditions (**Figure 4d**). For endophytic isolates, biofilm production remained stable regardless of osmotic stress (*p=0.230*), whereas rhizosphere isolates exhibited a significant reduction in biofilm capacity under drought (*p=0.047*). Notably, endophytes consistently produced significantly more biofilm than rhizosphere isolates under both non-stress (*p=0.011*) and drought conditions (*p=0.052*). Among the 42 biofilm-producing endophytes, *Pantoea dispersa* (Endo47) and *Pseudomonas mosselii* (Endo44) achieved the highest yields under non-stress and drought conditions, respectively (**Supplementary Table 4**). In the rhizosphere, although the group average decreased under stress, the number of active producers increased from 14 to 16, with *Serratia odorifera* (Rhizo9) reaching the maximum production (OD_560_ = 0.69) under drought (**Supplementary Table 5**).

Based on the clear zone, 20 endophyte isolates were able to solubilize phosphate, and nine isolates solubilized potassium (**Supplementary Figures 1a, b**; **Supplementary Table 4**). Of the rhizosphere strains, 8 were able to solubilize phosphate, and 3 isolated solubilized potassium (**Supplementary Table 5**). The isolate was also verified for the ability to produce siderophore, as known to release iron-chelating compounds. A total of 41 endophytic isolates and 18 rhizosphere isolates tested positive, producing prominent yellow halo zones on CAS agar plates (**Supplementary Figure 1c**; **Supplementary Tables 3**, **S5**). Furthermore, cellulose production was detected in 14 endophytic isolates and 6 rhizosphere isolates (**Supplementary Figure 1d**; **Supplementary Tables 4**, **S5**).

### 3.4 Comparative analysis of PGP traits of bacterial communities across cropping systems

**Figure 5:**
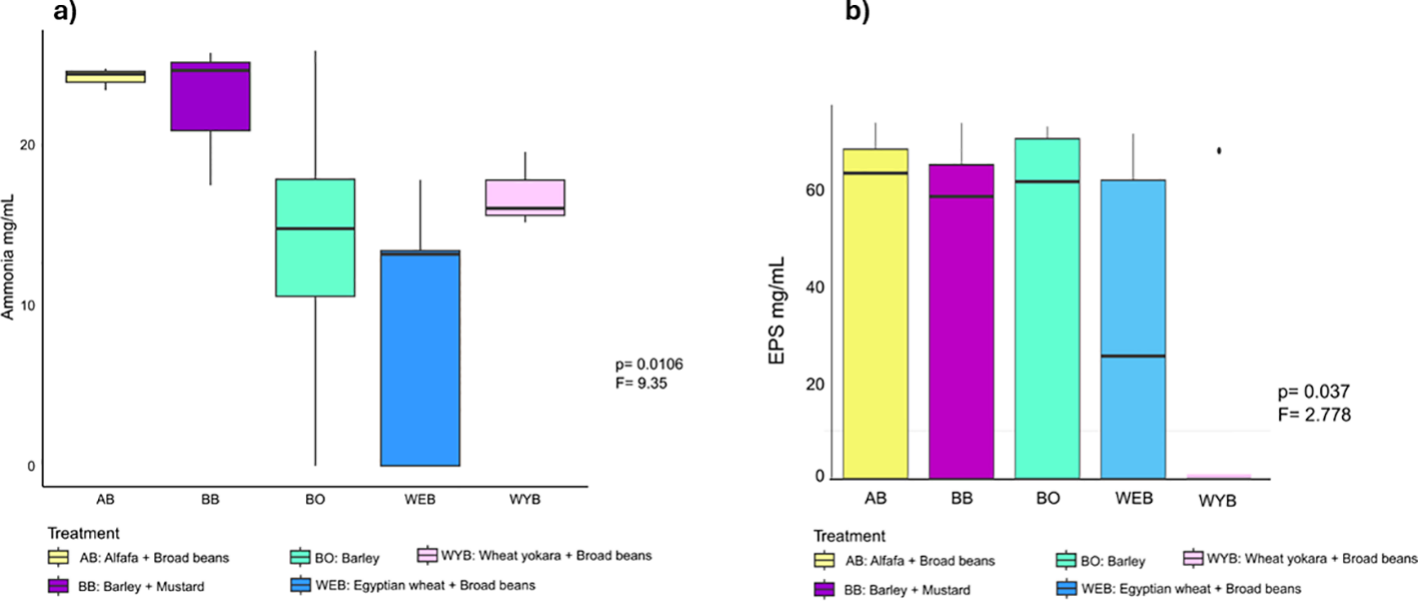
Comparative analysis of endophytic and rhizosphere isolates between the intercropping and monocropping systems. **(a)** Ammonia production by the rhizosphere isolates. **(b)** EPS production by the endophyte isolates. Bars represent mean mg/mL values ± standard deviation from triplicate experiments. Statistical differences among treatments were evaluated using one-way ANOVA followed by Tukey’s *post hoc* test. Statistical significance was considered *p < 0.05*. (Created with BioRender.com).

Comparative analysis revealed that the cropping system significantly influenced specific PGP traits, particularly N-fixation indicators and EPS production (**Figure 5**). Regarding N-fixation, rhizosphere isolates from the Egyptian wheat–broad bean treatment showed significantly different ammonia activity levels compared to those from alfalfa–broad bean (*p=0.017*) and barley–mustard (*p=0.010*) systems (**Figure 5a**). In contrast, no significant differences in ammonium production were observed among endophytic isolates across cropping systems.

Similarly, IAA production remained consistent across all treatments for both niches, regardless of L-tryptophan supplementation. For EPS production, a significant treatment effect was observed only in endophytic isolates when using glucose as the carbon source (F = 2.78, *p=0.037*; **Figure 5b**). *Post hoc* Tukey analysis confirmed that the Egyptian wheat–broad bean system differed significantly from the alfalfa–broad bean treatment (*p=0.047*). No other significant variations were found for EPS production in sucrose or among rhizosphere isolates. Furthermore, biofilm formation under both non-stress and drought conditions was not significantly affected by the cropping system (**Supplementary Figure 2**).

### 3.5 Assessment of the bacterial isolates’ potential to tolerate abiotic stress

**Figure 6:**
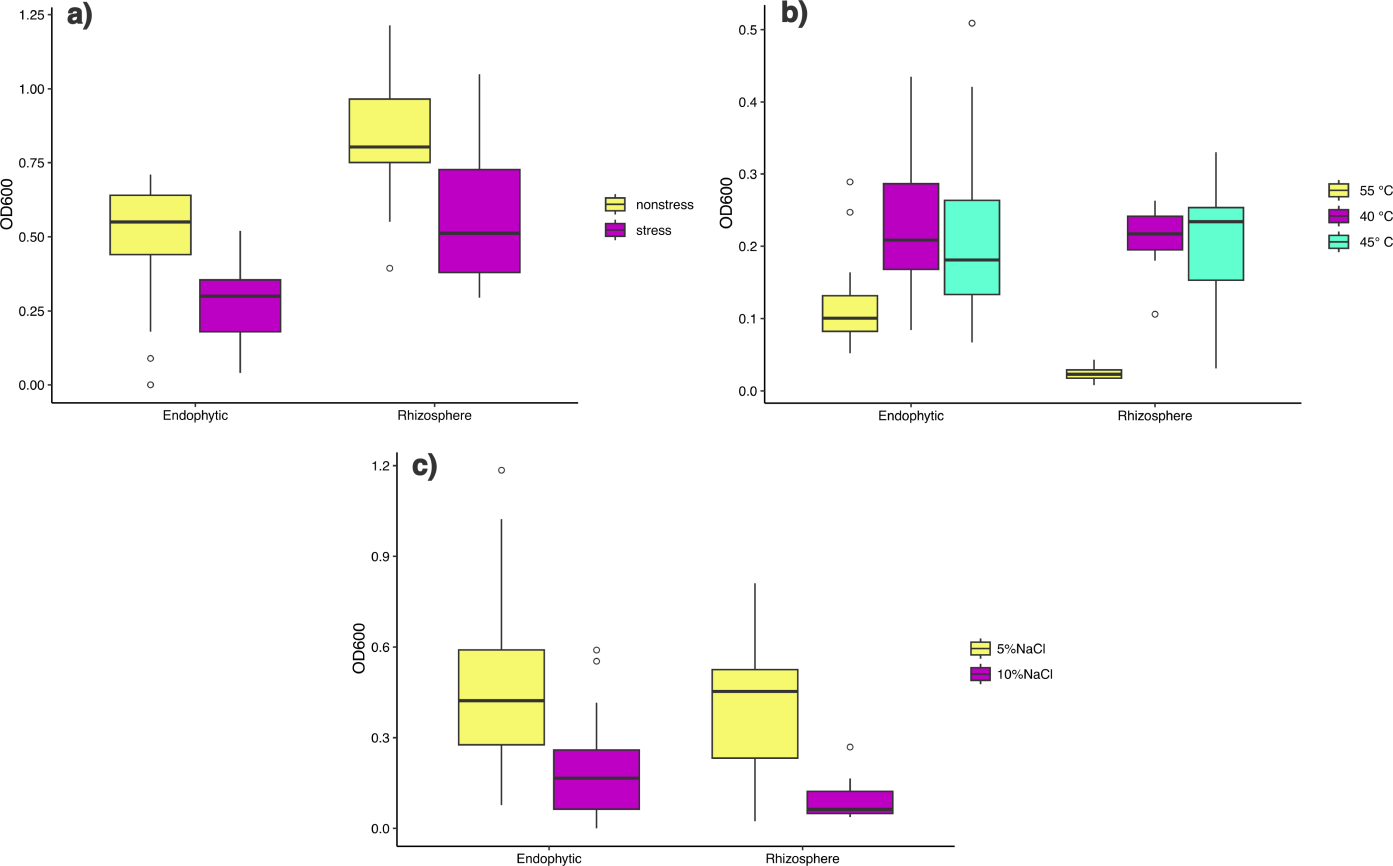
Growth of endophytic and rhizosphere isolates under drought, heat, and salinity stress conditions. **(a)** Drought stress was simulated with 10% PEG8000 in purple (-1.69 MPa osmotic potential) and under 0% PEG8000 in yellow. **(b)** heat stress at 40 °C (purple), 45 °C (green), and 55 °C (yellow). **(c)** Salinity stress with culture media supplemented with 5% (yellow) and 10% NaCl (purple). Bars represent mean OD_600_ values ± standard deviation from triplicate experiments. Statistical differences for drought and heat stress assays were evaluated using Welch’s two-sample t-test after testing for normality with the Shapiro–Wilk test, while salinity stress assays were analyzed using the Wilcoxon rank sum test (*p < 0.05*). (Created with BioRender.com).

Most isolates exhibited substantial tolerance to osmotic stress, with rhizosphere isolates consistently outperforming endophytic isolates under drought conditions (**Figure 6a**). Welch’s t-tests confirmed that rhizosphere isolates achieved significantly higher OD_600_ values compared to endophytes under drought-stress (0.582 vs. 0.267; *p<0.001*) conditions. All 55 endophytic isolates demonstrated growth under the tested osmotic potential (–1.69 MPa), with *Bacillus paramycoides* (Endo38) and *Pseudomonas simiae* (Endo33) showing the highest and lowest growth rates, respectively (**Supplementary Table 6**). Among the 25 rhizosphere isolates, 17 demonstrated significant growth (OD_600_ >0.1), led by *Bacillus subtilis* (Rhizo10; OD_600_ = 0.80), whereas *Morganella psychrotolerans* (Rhizo12) showed the minimum growth within this group (**Supplementary Table 7**).

The heat tolerance of the isolates varied significantly with temperature intensity (**Figure 6b**). Both endophytic and rhizosphere isolates exhibited similar growth patterns at 40 °C, with no significant differences observed (*p=0.350*) or 45 °C (*p=0.971*). However, endophytic isolates were markedly more resilient to extreme heat (55 °C) than rhizosphere isolates (0.111 vs. 0.023 OD_600_; *p<0.001*). A total of 46 endophytic isolates demonstrated growth across the entire temperature range, although a gradual decline in OD_600_ was noted as temperatures increased. The best performing endophytes included *Bacillus paralicheniformis* (Endo18) at 40 °C, *Pseudomonas siliginis* (Endo59) at 45 °C, and *Moraxella osloensis* (Endo13) at 55 °C (**Supplementary Table 6**). In contrast, while 13 rhizosphere isolates grew at 40 °C and 45 °C, led by Bacillus subtilis (Rhizo10), none exceeded the OD_600_ threshold of 0.1 at 55 °C, indicating a thermal limit for this group (**Supplementary Table 7**).

Both endophytic and rhizosphere isolates demonstrated the ability to grow under saline conditions (5% and 10% NaCl), although higher concentrations reduced overall viability (**Figure 6c**). While no significant difference was observed between the two groups at 5% NaCl (*p=0.298*), endophytic isolates exhibited significantly higher tolerance at 10% NaCl compared to rhizosphere isolates (*p=0.012*; Wilcoxon rank-sum test). Among endophytes at 5% NaCl, *Bacillus paralicheniformis* (Endo18) showed the highest growth (OD_600_ = 0.85). Interestingly, while most strains declined at 10% NaCl, *Achromobacter deleyi* (Endo9) and *Bacillus paranthracis* (Endo10) exhibited enhanced growth at the higher salinity level (OD_600_ of 0.18 and 0.59, respectively; **Supplementary Table 6**). In contrast, for the rhizosphere isolates, under 5% NaCl, the maximum OD_600_ value was 0.63 for strain Rhizo19 (*Proteus mirabilis*) and under 10% NaCl, most strains showed reduced growth, with the highest OD_600_ recorded at 0.17 for strain Rhizo23 (*Micrococcus yunnanensis*) (**Supplementary Table 7**).

### 3.6 Enzyme activity of endophytic and rhizosphere-associated bacteria

The ability to utilize ACC as a sole N source, indicating ACC deaminase production, was widespread among the isolates, being detected in 35 endophytic and 17 rhizosphere strains (**Supplementary Tables 8**, **S9**). Furthermore, most root-associated isolates exhibited a broad spectrum of extracellular enzymatic activities. Among endophytic isolates, xylanase and lipase were the most prevalent activities (83.82% and 80.88%, respectively), followed by amylase, cellulase, and protease, each present in approximately 79% of the strains (**Supplementary Table 8**). In the rhizosphere, a similar high prevalence was observed, with lipase (88.10%), protease (85.71%), and xylanase (85.50%) being the most common, followed by cellulase (81.40%) and amylase (80.10%) (**Supplementary Table 9**). Notably, only a few endophytic strains, including *Pseudomonas siliginis*, *Peribacillus castrilensis*, and *Pantoea agglomerans*, showed no detectable enzymatic activity under the tested conditions.

## 4 Discussion

Our study revealed distinct patterns in the composition of bacterial communities associated with different cropping systems in the UAE arid desert. *Pseudomonas*, *Bacillus*, and *Solibacillus* were identified as the dominant endophytic genera, while *Bacillus* was the most prevalent genus among rhizosphere isolates, followed by *Morganella* and *Proteus* (**Figure 2a**). These results highlight a selective recruitment of specific taxa, which is a common strategy for plants growing in arid environments to cope with abiotic stress (Wang et al., 2021). Interestingly, while the core genera remained consistent across treatments, their relative abundances varied according to the cropping system (**Figures 2b, c**). This suggests that although the arid environment exerts a strong pressure on microbial selection, the specific interaction between intercropping species further refines the assembly of both endophytic and rhizosphere communities. Variations in the endophytic and rhizosphere bacterial communities observed in our study may result from the combined effects of plant composition and soil characteristics. The soils in our study site are typical sandy soils (Shahid et al., 2013), characterized by drought and composed of > 90% sand, with low organic matter content (< 0.5%), and moderately alkaline pH values (7.5–8.5) (Pain et al., 2016). These harsh conditions, particularly low water availability and limited fertility, exert a strong selective pressure on the microbiota. In this context, the presence of specific bacterial genera across different cropping systems suggests that these microbes are well-adapted to the local conditions.

In arid regions, the combination of intercropping systems and PGPB represents a promising strategy to enhance agricultural productivity and reduce dependence on food imports. Our results revealed differences in functional traits between the isolates from cropping systems. In particular, some isolates from intercropping plants enhanced IAA and EPS production, and drought tolerance (**Figures 5a, b**), suggesting that crop diversification may favor the recruitment of functionally diverse bacteria under arid conditions. These findings align with previous reports showing that diverse cropping systems can shape root-associated microbial communities by promoting the selection of bacteria with enhanced metabolic capabilities (Zhou et al., 2023; Jiang et al., 2024). The predominance of bacterial isolates exhibiting stress-related traits highlights their potential ecological role in arid environments, where water availability represents a major limitation for plant growth. Given the importance of identifying effective PGPB for drylands, our study focused on isolates capable of mitigating nutrient limitations. All isolates indicated the ability to fix N, a key mechanism for supporting crops in nutrient-poor soils. While phosphorus and potassium are often present in insoluble forms in arid lands, necessitating high fertilizer inputs (Dey et al., 2021), our findings revealed that specific intercropping combinations can favor the recruitment of solubilizing bacteria. For instance, Egyptian wheat intercropped with broad beans showed a higher frequency of phosphate-solubilizing rhizosphere isolates, whereas barley with mustard and wheat yokara with broad beans favored endophytic phosphate solubilization (**Supplementary Tables 4**, **S5**). Regarding potassium solubilization, wheat yokara and broad bean combination stood out, harboring more endophytic isolates with this trait compared to other systems. These variations in nutrient-solubilizing traits may reflect differences in the metabolic capabilities of bacterial isolates, including the production of extracellular organic acids involved in mineral solubilization (Raji and Thangavelu, 2021).

In addition to macronutrient availability, the acquisition of essential micronutrients like iron (Fe) is a critical challenge in agricultural soils, especially in arid sites where the availability of metal nutrients, particularly Fe, drops dramatically. Under these alkaline and water-limited conditions, Fe often exists in the non-bioavailable Fe state (Fe3+), limiting plant development (Tang et al., 2019). Our findings showed that intercropping systems, particularly the combination of barley and mustard, favored a higher frequency of siderophore-producing isolates in both endophytic and rhizosphere niches (**Supplementary Tables 4**, **S5**). This enhanced production of low-molecular-weight chelators acts as a synergistic mechanism for mobilizing and delivering Fe to the plant while simultaneously regulating its internal concentration to avoid toxicity (Sultana et al., 2021). Furthermore, the prevalence of these isolates in the intercropping treatments may offer dual benefits: improving Fe nutrition and providing biological control against potential pathogens (Sun et al., 2022). These results suggest that intercropping-associated bacteria, such as those from the *Bacillus* and *Pseudomonas* genera identified in our study, may play a multifaceted role in plant fitness under adverse conditions.

Beyond metabolic mobilization, the production of IAA is a key trait that directly influences root architecture and nutrient acquisition (Grover et al., 2021). In our study, all isolates (endophytic and rhizosphere niches across all cropping systems) demonstrated the capacity to produce IAA (**Figure 4b**). This widespread PGP trait suggests a conserved mechanism for plant growth stimulation among the bacteria adapted to the arid desert conditions of the UAE, like observations in native plants from the Saudi Arabian desert (Eida et al., 2018). Furthermore, the functional versatility of these isolates was evidenced by the production of intracellular and extracellular enzymes, which play critical roles in nutrient cycling and plant-microbe interactions (Ali et al., 2024; Shu et al., 2024). Notably, endophytic isolates exhibited a significantly higher enzymatic potential compared to rhizosphere isolates, particularly for xylanase (85.50%), protease (85.71%), and lipase (88.10%) (**Supplementary Tables 8**, **S9**). Similar multifunctional profiles have been reported in *Bacillus* spp. from stressed environments, reinforcing the potential of these bacteria to maintain metabolic activity under adverse conditions (Lee et al., 2024; Ayaz et al., 2024). This resilience is further supported by the production of ACC deaminase, which was enhanced in isolates from the barley and mustard intercropping system. By cleaving the ethylene precursor (ACC) into ammonia and α-ketobutyrate, this enzyme prevents stress-induced growth inhibition, thereby facilitating plant-microbe interactions and survival in arid soils (Moon and Ali, 2022).

Under environmental stress, bacteria adopt strategies such as the production of EPS and biofilms to enhance water retention and nutrient acquisition (Ajijah et al., 2023). Our results showed that most endophytic isolates preferentially produced EPS when sucrose was used as a carbon source, whereas the carbon source did not significantly affect production in rhizosphere isolates (**Figure 4c**). Since EPS production is influenced by carbon metabolism and substrate availability (Fuso et al., 2023), these results suggest distinct physiological responses between endophytic and rhizosphere bacterial communities. Furthermore, the ability of most strains to form biofilms under osmotic stress (-1.69 MPa) reinforces their potential to create hydrated microenvironments that support plant survival under drought conditions (Raklami et al., 2024). The biofilm formation and enhanced cellulose production observed in our isolates likely facilitate root surface attachment and protective agglutinating activity. Regarding abiotic tolerance, most isolates in our study demonstrated tolerance to drought, heat, and salinity stress, supporting their survival strategy under these conditions. In this context, these microorganisms can develop intimate interactions with plants to achieve an adequate strategy for survival.

## 5 Conclusion

This study demonstrates that intercropping systems in arid regions promote a diverse and functionally versatile bacterial microbiome in both endophytic and rhizosphere niches. The major findings indicate that isolates from these systems, particularly Bacillus and Pseudomonas genera, possess multiple PGP traits, including N-fixation, nutrient solubilization, and significant IAA and EPS production. Notably, endophytic isolates exhibited superior enzymatic potential and niche-specific metabolic adaptations compared to rhizosphere isolates. The practical implications of these findings for arid agriculture are significant: the identified PGPB strains, capable of withstanding extreme osmotic stress (-1.69 MPa) and high temperatures (45 °C), represent promising candidates for the development of biofertilizers. Such biotechnological solutions could reduce reliance on chemical fertilizers and improve crop resilience in water-limited environments. Future research should focus on field-scale trials to evaluate the synergistic effects of these bacterial consortia on plant physiology and to validate the species-specific benefits of intercropping through comparative monoculture assessments.

## Data Availability

Sanger sequence data generated in this study are available at NCBI with accession number: PZ548039 - PZ548118.
