# Supplementary material for: Distinct functional responses of root endophyte and rhizosphere microbial communities in intercropping systems under arid conditions
Source: Front Plant Sci. 2026 Jun 30;17:1809801. doi: 10.3389/fpls.2026.1809801 (PMC13364846; doi:10.3389/fpls.2026.1809801)
Supplement: Supplementary file 1 [file DataSheet1.docx]

***Distinct functional responses of root and rhizosphere microbial communities in intercropping systems under arid conditions***

Sulamita Santos Correa^1^; Aysha Mohammed Aksayan Almansoori^1^; Shahnaz Nazzar^1,3^; Balamurugan Sadaiappan^2^; Qurban Ali^1^; Ponnarmadha Subramani^1^; Sunil Mundra^1,2,*^

^1^Department of Biology, College of Science, United Arab Emirates University, Al Ain, United Arab Emirates

^2^Khalifa Center for Genetic Engineering and Biotechnology, United Arab Emirates University, United Arab Emirates.

^3^School of Biology, Indian Institute of Science Education and Research, Thiruvananthapuram.

***Corresponding author:** [sunilmundra@uaeu.ac.ae](mailto:sunilmundra@uaeu.ac.ae) (Sunil Mundra)

**Table S1.** Culture medium description.

| Culture medium | Reagents | Manufacture | Reference |
| --- | --- | --- | --- |
| Luria Bertani (LB) Agar medium | Agar, 15 g/L NaCl, 10 g/L Tryptone, 10 g/L Yeast Extract, 5 g/L | Sigma-Aldrich | de Jesus Bertani, A. M., Vieira, T., Reis, A. D., Dos Santos, C. A., de Almeida, E. A., Camargo, C. H., & Casas, M. R. T. (2023). Whole genome sequence analysis of the first reported isolate of Salmonella Agona carrying blaCTX-M-55 gene in Brazil. *Scientific Reports*, *13*(1), 2299. |
| Nutrient Agar (NA) medium | Agar, 15.0 g/L Peptone (vegetable), 5.0 g/L Vegetable extract, 3.0 g/L | Millipore | Chhetri, S., Sherpa, M. T., & Sharma, L. (2025). Characterization of plant growth promoting bacteria isolated from rhizosphere of tomato cultivated in Sikkim Himalaya and their potential use as biofertilizer. *Scientific Reports*, *15*(1), 15558. https://doi.org/10.1038/s41598-025-98953-6 |
| Nutrient Broth (NB) medium | D(+)-glucose, 1 g/L Peptone, 15 g/L Sodium chloride, 6 g/L Yeast extract, 3 g/L | Millipore | Chhetri, S., Sherpa, M. T., & Sharma, L. (2025). Characterization of plant growth promoting bacteria isolated from rhizosphere of tomato cultivated in Sikkim Himalaya and their potential use as biofertilizer. *Scientific Reports*, *15*(1), 15558. https://doi.org/10.1038/s41598-025-98953-6 |
| Jensen's medium | Sucrose 20, g/L  Dipotassium hydrogen phosphate, 1 g/L  Magnesium sulphate, 0.5 g/L  Sodium chloride, 0.5 g/L  Ferrous sulphate, 0.1 g/L  Sodium molybdate, 0.005 g/L  Calcium carbonate, 2 g/L  Agar, 15 g/L | Himedia | Yan, D., Tajima, H., Cline, L. C., Fong, R. Y., Ottaviani, J. I., Shapiro, H. Y., & Blumwald, E. (2022). Genetic modification of flavone biosynthesis in rice enhances biofilm formation of soil diazotrophic bacteria and biological nitrogen fixation. *Plant Biotechnology Journal*, *20*(11), 2135-2148. |
| Peptone water medium | Peptone, 10 g/L Sodium chloride, 5 g/L | Millipore | Nithyapriya, S., Lalitha, S., Sayyed, R. Z., Reddy, M. S., Dailin, D. J., El Enshasy, H. A., ... & Herlambang, S. (2021). Production, purification, and characterization of bacillibactin siderophore of Bacillus subtilis and its application for improvement in plant growth and oil content in sesame. *Sustainability*, *13*(10), 5394. |
| Yeat Mannitol (YM) medium | Yeast extract, 0,4 g/L Mannitol, 10 g/L Dipotassium phosphate 10%, 1 mL  Monopotassium phosphate 10%, 4 mL Magnesium sulfate 10%, 2 mL Sodium chloride 10%, 1 mL  Bromothymol blue 0,5%, 5 mL Agar, 15.00  Sucrose 10%  Or Glucose 10% |  | Correa, S. S., Pacheco, R. S., Viana, G. C., Vidal, M. S., Xavier, G. R., & de Araújo, J. L. S. (2024). Ability of nitrogen-fixing bacteria to alleviate drought stress in cowpea varies depending on the origin of the inoculated strain. *Plant and Soil*, *498*(1), 391-408. https://doi.org/10.1007/s11104-023-06443-3 |
| Glucose-Yeast Ethanol Extract Agar (GYEA) | Glucose, 20 g/L  Yeast extract, 10 g/L  Ethanol (absolute), 50 mL  Calcium carbonate, 3 g/L  Agar, 20 g/L |  | Lee, S., Park, M. S., Lee, H., Kim, J. J., Eimes, J. A., & Lim, Y. W. (2019). Fungal diversity and enzyme activity associated with the macroalgae, Agarum clathratum. *Mycobiology*, *47*(1), 50-58. |
| Pikovskaya’s agar medium | Yeast extract, 0,5 g/L  Glucose, 10 g/L  Calcium phosphate, 5 g/L  Ammonium sulphate, 0,5 g/L  Potassium chloride, 0,2 g/L  Magnesium sulphate, 0,1 g/L  Manganese sulphate, 0,0001 g/L  Ferrous sulphate, 15 g/L  Agar, 15 g/L | Himedia | Sanchez-Gonzalez, M. E., Mora-Herrera, M. E., Wong-Villarreal, A., De La Portilla-López, N., Sanchez-Paz, L., Lugo, J., ... & Yañez-Ocampo, G. (2022). Effect of pH and carbon source on phosphate solubilization by bacterial strains in pikovskaya medium. *Microorganisms*, *11*(1), 49. https://doi.org/10.3390/microorganisms11010049 |
| Aleksandrow agar medium | Magnesium sulphate, 0,5 g/L  Calcium carbonate, 0,1 g/L  Potassium alumino silicate, 2 g/L  Glucose, 5 g/L  Ferric chloride, 0,005 g/L  Calcium phosphate, 2 g/L  Agar, 20 g/L | Himedia | Raji, M., & Thangavelu, M. (2021). Isolation and screening of potassium solubilizing bacteria from saxicolous habitat and their impact on tomato growth in different soil types. *Archives of Microbiology*, *203*(6), 3147-3161. https://doi.org/10.1007/s00203-021-02284-9 |
| DF minimal medium  Trace elements | Monopotassium phosphate, 4 g/L  Disodium hydrogen phosphate, 6 g/L  Magnesium sulphate, 0.2 g/L  Glucose, 2 g/L  Gluconic acid, 2 g/L  Citric acid, 2 g/L  Iron sulfate, 1 mg/L  Boric acid, 10 mg/L  Manganese sulfate, 11.19 mg/L  Zinc sulfate, 124.6 mg/L  Copper sulfate, 78.22 mg/L  Molybdenum trioxide, 10 mg/L  Agar, 20 g/L |  | Penrose, D. M., & Glick, B. R. (2003). Methods for isolating and characterizing ACC deaminase‐containing plant growth‐promoting rhizobacteria. *Physiologia plantarum*, *118*(1), 10-15. https://doi.org/10.1034/j.1399-3054.2003.00086.x |

**Table S2.** Envfit analyses results for different vector and factors while NMDS ordination analyses.

|  | gnmds1 | gnmds2 | r2 | Pr(>r) |  |
| --- | --- | --- | --- | --- | --- |
| N_fixation | 0.52529 | 0.85092 | 0.029 | 0.337 |  |
| ACC.deaminase | 0.95152 | -0.30758 | 0.007 | 0.756 |  |
| Siderophore | -0.34522 | -0.93852 | 0.0332 | 0.274 |  |
| P_Solubilization | 0.26898 | -0.96314 | 0.0017 | 0.936 |  |
| K_Solubilization | 0.4356 | 0.90014 | 0.0122 | 0.624 |  |
| EPS_Glusose | 0.92176 | -0.38777 | 0.1058 | **0.013** | * |
| EPS_Sucrose | -0.0256 | -0.99967 | 0.0242 | 0.403 |  |
| Sucrose_EPS | -0.96827 | 0.24991 | 0.3752 | **0.001** | *** |
| Glucose_EPS | 0.36644 | 0.93044 | 0.6178 | **0.001** | *** |
| Biofilm | -0.86224 | 0.5065 | 0.0322 | 0.287 |  |
| Biofilm_stress | -0.87148 | 0.49044 | 0.0703 | 0.068 | . |
| IAA | -0.77988 | 0.62593 | 0.5004 | **0.001** | *** |
| IAA_Trip | -0.87982 | 0.47532 | 0.4425 | **0.001** | *** |
| Ammonia | 0.98398 | -0.17828 | 0.0783 | 0.058 | . |
| Non_drought | -0.60101 | 0.79924 | 0.0194 | 0.471 |  |
| Drought_stress | 0.98553 | 0.16952 | 0.126 | **0.006** | ** |
| Temperature 40C | -0.50737 | 0.86173 | 0.0351 | 0.278 |  |
| Temperature 45C | -0.7747 | 0.63233 | 0.064 | 0.085 | . |
| Temperature 55C | -1 | 0.00082 | 0.0803 | **0.035** | * |
| 5% NaCl | 0.16886 | 0.98564 | 0.0159 | 0.542 |  |
| 10% NaCl | -0.99825 | -0.0592 | 0.0698 | 0.071 | . |
| Crop_species |  |  | 0.0564 | 0.384 |  |
| Origin (Endosphere vs Rhizosphere) |  |  | 0.1991 | **0.001** |  |

**Table S3:** Combined results of plant species, biochemical traits and taxonomical of isolated endophytic strains. The values represent the mean ± standard deviation of three replicates. (+) indication of no production; (-) indication of no production.

| Isolates | Taxonomic | Intercropping | Ammonia  production mg/mL | EPS Sucrose mg/mL | EPS Glucose mg/mL | Biofilm production OD_600_ | Biofilm  10% PEG OD_600_ | IAA not L-trp  μg/ml | IAA L-trp  μg/ml |
| --- | --- | --- | --- | --- | --- | --- | --- | --- | --- |
| Endo1 | *Solibacillus silvestres* | BB | 8,554 ± 0,03 | - | - | 0,11 ± 0,06 | 0,22 ± 0,06 | 125,21±0,012 | 372,29 ± 0,013 |
| Endo2 | *Kosakonia cowanii* | BO | 13,18 ± 0,05 | 80,79 ± 0,01 | 70,91 ± 0,02 | 0,1 ± 0,02 | 0,1 ± 0,07 | 99,79 ± 0,026 | 163,63 ± 0,04 |
| Endo3 | *Pseudomonas mosselii* | WYB | 8,774 ± 0,02 | 86,32 ± 0,02 | - | 0,22 ± 0,03 | 0,49 ± 0,11 | 91,88 ± 0,004 | 299,79 ± 0,02 |
| Endo4 | *Solibacillus isronensis* | WYB | 8,023 ± 0,06 | 81,39 ± 0,32 | - | 0,13 ± 0,12 | 0,18 ± 0,12 | 58,96 ± 0,003 | 175,63 ± 0,05 |
| Endo5 | *Pseudomonas argentinensis* | BO | 14,158 ± 0,14 | 86,46 ± 0,03 | - | 0,15 ± 0,08 | 0,21 ± 0,05 | 77,71 ± 0,001 | 336,04 ± 0,10 |
| Endo6 | *Bacillus paranthracis* | AB | 12,382 ± 0,01 | 83,52 ± 0,01 | - | 0,17 ± 0,02 | 0,41 ± 0,09 | 121,88 ± 0,012 | 391,46 ± 0,18 |
| Endo7 | *Pantoea agglomerans* | BO | 13,371 ± 0,07 | 91,72 ± 0,03 | 73,47 ± 0,04 | 1,22 ± 0,33 | 0,79 ± 0,13 | 120,63 ± 0,002 | 84,79 ± 0,01 |
| Endo8 | *Pseudomonas granadensis* | BB | 14,799 ± 0,09 | 77,52 ± 0,17 | 62,3 ± 0,17 | 0,07 ± 0,01 | 0,19 ± 0,07 | 106,88 ± 0,013 | 126,46 ± 0,01 |
| Endo9 | *Achromobacter deleyi* | WYB | 12,949 ± 0,00 | 90,05 ± 0,03 | 68,41 ± 0,04 | 0,09 ± 0,05 | - | 135,63 ± 0,006 | 88,13 ± 0,01 |
| Endo10 | *Bacillus paranthracis* | BO | 13,169 ± 0,00 | 87,39 ± 0,02 | 56,08 ± 0,09 | 0,09 ± 0,03 | 0,39 ± 0,45 | 154,38 ± 0,010 | 147,71 ± 0,01 |
| Endo11 | *Solibacillus isronensis* | AB | 10,99 ± 0,08 | 78,79 ± 0,12 | - | 0,15 ± 0,07 | - | 81,88 ± 0,002 | 90,21 ± 0,00 |
| Endo12 | *Pantoea agglomerans* | WEB | 14,854 ± 0,06 | 80,72 ± 0,24 | 62,25 ± 0,16 | 0,17 ± 0,17 | 0,67 ± 0,08 | 56,04 ± 0,003 | 127,71 ± 0,02 |
| Endo13 | *Moraxella osloensis* | BB | 3,803 ± 0,05 | 86,25 ± 0,04 | - | 0,12 ± 0,02 | - | 79,37 ± 0,013 | 64,79 ± 0,01 |
| Endo14 | *Achromobacter deleyi* | BO | 6,649 ± 0,03 | 88,99 ± 0,06 | - | 0,13 ± 0,00 | 0,17 ± 0,03 | 74,38 ± 0,007 | 59,79 ± 0,01 |
| Endo15 | *Enterobacter kobei* | AB | 16,081 ± 0,07 | 4,43 ± 0,05 | 74,25 ± 0,11 | 0,18 ± 0,06 | 0,46 ± 0,11 | 84,37 ± 0,008 | 158,13 ± 0,02 |
| Endo16 | *Peribacillus frigoritolerans* | AB | 11,686 ± 0,02 | 86,59 ± 0,06 | 65,25 ± 0,23 | - | 0,49 ± 0,34 | 78,13 ± 0,003 | 68,96 ± 0,00 |
| Endo18 | *Bacillus paralicheniformis* | WEB | 13,536 ± 0,04 | 85,54 ± 0,38 | - | 0,09 ± 0,03 | 0,58 ±0,12 | 128,54 ± 0,011 | 100,63 ± 0,01 |
| Endo19 | *Bacillus paranthracis* | AB | 12,84 ± 0,07 | 82,19 ± 0,01 | 73,69 ± 0,06 | 0,04 ± 0,01 | - | 170,21 ± 0,007 | 93,54 ± 0,03 |
| Endo20 | *Pseudomonas mosselii* | BB | 9,323 ± 0,14 | 84,32 ± 0,02 | 74,19 ± 0,05 | 0,12 ± 0,06 | 0,19 ± 0,03 | 98,96 ± 0,005 | 90,21 ± 00 |
| Endo22 | *Solibacillus isronensis* | BB | 6,521 ± 0,06 | 89,52 ± 0,03 | 65,47 ± 0,22 | 0,11 ± 0,05 | 0,24 ± 0,14 | 97,71 ± 0,012 | 125,63 ± 0,01 |
| Endo23 | *Kocuria sediminis* | BB | 5,074 ± 0,03 | 80,72 ± 0,26 | 69,91 ± 0,08 | 0,16 ± 0,12 | - | 113,13 ± 0,000 | 98,54 ± 0,00 |
| Endo24 | *Pseudomonas shirazensis* | BB | 7,437 ± 0,09 | 82,19 ± 0,06 | 73,86 ± 0,01 | 1,32 ± 0,08 | 0,33 ± 0,03 | 189,79 ± 0,019 | 161,88 ± 0,02 |
| Endo26 | *Enterobacter kobei* | AB | 8,554 ± 0,01 | 83,79 ± 0,01 | 62,20 ± 0,01 | 0,57 ± 0,11 | 0,74 ± 0,05 | 193,54 ± 0,015 | 136,46 ± 0,01 |
| Endo27 | *Pseudoroseomonas rhizosphaerae* | BO | 19,763 ± 0,02 | - | 61,97 ± 0,16 | 0,12 ± 0,07 | 0,24 ± 0,05 | 91,88 ± 0,013 | 183,96 ± 0,11 |
| Endo28 | *Peribacillus castrilensis* | AB | 9,689 ± 0,03 | 79,85 ± 0,24 | 66,19 ± 0,02 | 0,74 ± 0,37 | 0,18 ± 0,04 | 139,79 ± 0,018 | 394,38 ± 0,21 |
| Endo29 | *Pseudomonas granadensis* | BB | 13,114 ± 0,02 | 83,92 ± 0,08 | - | 0,1 ± 0,03 | 0,22 ± 0,09 | 107,71 ± 0,013 | 133,13 ± 0,03 |
| Endo30 | *Lysinibacillus odysseyi* | BO | 9,048 ± 0,04 | 85,05 ± 0,03 | - | 0,14 ± 0,08 | 0,3 ± 0,10 | 99,38 ± 0,013 | 110,63 ± 0,02 |
| Endo32 | *Pseudomonas mendocina* | BB | 14,47 ± 0,02 | 87,72 ± 0,04 | - | 0,17 ± 0,07 | 3,9 ± 0,10 | 69,79 ± 0,005 | 166,88 ± 0,01 |
| Endo33 | *Pseudomonas simiae* | WEB | 15,367 ± 0,08 | 81,85 ± 0,24 | - | 0,33 ± 0,17 | 0,08 ± 0,01 | 81,46 ± 0,019 | 113,96 ± 0,00 |
| Endo35 | *Staphylococcus succinus* | BB | 12,052 ± 0,01 | 86,99 ± 0,06 | - | 1,08 ± 0,56 | 0,18 ± 0,10 | 84,37 ± 0,051 | 30,21 ± 0,01 |
| Endo38 | *Bacillus paramycoides* | BO | 13,554 ± 0,09 | 80,79 ± 0,21 | 71,86 ± 0,01 | 0,09 ± 0,04 | 0,06 ± 0,01 | 137,29 ± 0,021 | 27,29 ± 0,00 |
| Endo39 | *Pseudomonas mosselii* | WYB | 18,719 ± 0,06 | 85,45 ± 0,02 | - | 0,45 ± 0,02 | 0,67 ± 0,09 | 49,79 ± 0,008 | 214,38 ± 0,06 |
| Endo40 | *Flavobacterium hibisci* | AB | 10,678 ± 0,03 | - | 72,02 ± 0,08 | 0,08 ± 0,01 | 0,12 ± 0,04 | 69,38 ± 0,013 | 86,46 ± 0,01 |
| Endo42 | *Lysinibacillus odysseyi* | WYB | 16,155 ± 0,11 | - | - | 0,04 ± 0,01 | 0,23 ± 0,06 | 53,13 ± 0,021 | 39,38 ± 0,01 |
| Endo44 | *Pseudomonas mosselii* | AB | 15,202 ± 0,09 | 82,52 ± 0,31 | 67,63 ± 0,06 | 0,39 ± 0,09 | 1,4 ± 0,09 | 131,04 ± 0,023 | 81,88 ± 0,01 |
| Endo45 | *Solibacillus isronensis* | BB | 12,473 ± 0,05 | 87,52 ± 0,01 | 65,36 ± 0,04 | 0,84 ± 0,16 | 0,54 ± 0,10 | 61,46 ± 0,016 | 82,29 ± 0,01 |
| Endo47 | *Pantoea dispersa* | BB | 15,376 ± 0,04 | 86 ± 0,02 | - | 1,33 ± 0,11 | 0,84 ± 0,10 | 73,96 ± 0,025 | 56,46 ± 0,01 |
| Endo48 | *Lysinibacillus capsici* | BB | 10,807 ± 0,02 | 40,19 ± 0,15 | 67,08 ± 0,04 | - | - | 81,46 ± 0,016 | 45,21 ± 0,01 |
| Endo49 | *Lysinibacillus odysseyi* | WYB | 12,895 ± 0,07 | 91,65 ± 0,06 | - | - | - | 83,54 ± 0,014 | 91,88 ± 0,00 |
| Endo50 | *Pseudomonas mosselii* | WEB | 16,338 ± 0,08 | 85,45 ± 0,09 | 25,58 ± 0,02 | 0,15 ± 0,05 | - | 36,46 ± 0,007 | 93,96 ± 0,00 |
| Endo51 | *Kosakonia cowanii* | BO | 14,177 ± 0,02 | 74,39 ± 0,17 | 64,75 ± 0,25 | - | - | 26,88 ± 0,008 | 57,71 ± 0,01 |
| Endo54 | *Exiguobacterium indicum* | AB | 16,759 ± 0,05 | - | - | 0,06 ± 0,03 | - | 45,63 ± 0,011 | 95,63 ± 0,02 |
| Endo55 | *Pseudomonas mosselii* | BB | 18,352 ± 0,09 | 84,19 ± 0,38 | 58,86 ± 0,13 | - | - | 36,88 ± 0,007 | 68,54 ± 0,00 |
| Endo56 | *Pseudomonas benzopyrenica* | BB | 10,752 ± 0,06 | 88,05 ± 0,03 | 64,3 ± 0,02 | 0,14 ± 0,10 | - | 47,29 ± 0,010 | 118,13 ± 0,01 |
| Endo57 | *Peribacillus castrilensis* | WYB | 11,21 ± 0,07 | - | - | - | - | 37,71 ± 0,005 | 65,54 ± 0,01 |
| Endo58 | *Lysinibacillus odysseyi* | BB | 13,481 ± 0,01 | 91,45 ± 0,15 | 52,41 ± 0,04 | - | - | 189,79 ± 0,052 | 59,38 ± 0,00 |
| Endo59 | *Pseudomonas siliginis* | AB | 13,627 ± 0,06 | 77,45 ± 0,17 | 42,3 ± 0,04 | - | - | 124,79 ± 0,008 | 116,88 ± 0,01 |
| Endo60 | *Pseudomonas siliginis* | BB | 15,203 ± 0,05 | 88,52 ± 0,03 | - | - | 0,06 ± 0,02 | 54,38 ± 0,005 | 74,79 ± 0,00 |
| Endo61 | *Pseudomonas simiae* | WYB | 13,261 ± 0,05 | 89,59 ± 0,07 | - | - | - | 51,04 ± 0,041 | 294,79 ± 0,26 |
| Endo62 | *Pseudomonas iranensis* | AB | 13,829 ± 0,03 | 89,59 ± 0,09 | - | - | - | 162,71 ± 0,017 | 145,63 ± 0,03 |
| Endo63 | *Solibacillus isronensis* | WYB | 7,144 ± 0,02 | 82,45 ± 0,30 | - | - | - | 21,88 ± 0,015 | 94,38 ± 0,01 |
| Endo64 | *Solibacillus isronensis* | BB | 5,733 ± 0,03 | - | - | - | 0,06 ± 0,03 | 66,88 ± 0,016 | 76,88 ± 0,03 |
| Endo65 | *Bacillus paralicheniformis* | WEB | 15,111 ± 0,07 | - | 71,97 ± 0,02 | 0,04 ± 0,01 | 0,27 ± 0,07 | 118,54 ± 0,012 | 122,71 ± 0,01 |
| Endo66 | *Bacillus anthracis* | WYB | 14,579 ± 0,05 | 86,59 ± 0,07 | - | 0,12 ± 0,03 | 0,77 ± 0,45 | 235,21 ± 0,085 | 24,79 ± 0,00 |
| Endo67 | *Bacillus paramycoides* | WYB | 12,931 ± 0,06 | 82,52 ± 0,30 | - | 0,13 ± 0,02 | 0,14 ± 0,03 | 54,38 ± 0,012 | 53,54 ± 0,00 |
|  |  |  |  |  |  |  |  |  |  |
| Isolates | Taxonomic | Intercropping | Nitrogen fixation | EPS glucose | EPS sucrose | P solubilization | K solubilization | Siderophore | Cellulose |
| Endo1 | *Solibacillus silvestres* | BB | *+* | - | - | - | - | - | - |
| Endo2 | *Kosakonia cowanii* | BO | *+* | + | + | + | - | + | + |
| Endo3 | *Pseudomonas mosselii* | WYB | *+* | - | + | + | + | + | - |
| Endo4 | *Solibacillus isronensis* | WYB | *+* | - | + | + | - | + | - |
| Endo5 | *Pseudomonas argentinensis* | BO | *+* | - | + | - | - | + | - |
| Endo6 | *Bacillus paranthracis* | AB | *+* | - | + | - | - | + | + |
| Endo7 | *Pantoea agglomerans* | BO | *+* | + | + | - | - | + | - |
| Endo8 | *Pseudomonas granadensis* | BB | *+* | + | + | - | - | + | + |
| Endo9 | *Achromobacter deleyi* | WYB | *+* | + | + | - | + | + | + |
| Endo10 | *Bacillus paranthracis* | BO | *+* | + | + | - | - | + | - |
| Endo11 | *Solibacillus isronensis* | AB | *+* | - | + | + | - | + | * |
| Endo12 | *Pantoea agglomerans* | WEB | *+* | + | + | + | - | - | * |
| Endo13 | *Moraxella osloensis* | BB | *+* | - | + | - | - | + | - |
| Endo14 | *Achromobacter deleyi* | BO | *+* | - | + | - | - | + | - |
| Endo15 | *Enterobacter kobei* | AB | *+* | + | + | + | - | + | + |
| Endo16 | *Peribacillus frigoritolerans* | AB | *+* | + | + | - | - | + | - |
| Endo18 | *Bacillus paralicheniformis* | WEB | *+* | - | + | - | - | + | - |
| Endo19 | *Bacillus paranthracis* | AB | *+* | + | + | - | - | - | - |
| Endo20 | *Pseudomonas mosselii* | BB | *+* | + | + | + | - | + | - |
| Endo22 | *Solibacillus isronensis* | BB | *+* | + | + | - | - | + | - |
| Endo23 | *Kocuria sediminis* | BB | *+* | + | + | - | - | + | - |
| Endo24 | *Pseudomonas shirazensis* | BB | *+* | + | + | - | - | + | + |
| Endo26 | *Enterobacter kobei* | AB | *+* | + | + | + | - | - | - |
| Endo27 | *Pseudoroseomonas rhizosphaerae* | BO | *+* | + | - | - | + | - | - |
| Endo28 | *Peribacillus castrilensis* | AB | *+* | + | + | - | - | + | - |
| Endo29 | *Pseudomonas granadensis* | BB | *+* | - | + | - | - | + | - |
| Endo30 | *Lysinibacillus odysseyi* | BO | *+* | - | + | - | + | + | - |
| Endo32 | *Pseudomonas mendocina* | BB | *+* | - | + | - | - | + | - |
| Endo33 | *Pseudomonas simiae* | WEB | *+* | - | + | + | - | + | + |
| Endo35 | *Staphylococcus succinus* | BB | *+* | - | + | - | -- | + | + |
| Endo38 | *Bacillus paramycoides* | BO | *+* | + | + | - | - | - | - |
| Endo39 | *Pseudomonas mosselii* | WYB | *+* | - | + | + | + | - | + |
| Endo40 | *Flavobacterium hibisci* | AB | *+* | + | - | - | - | + | - |
| Endo42 | *Lysinibacillus odysseyi* | WYB | *+* | - | - | - | - | * | - |
| Endo44 | *Pseudomonas mosselii* | AB | *+* | + | + | + | + | - | - |
| Endo45 | *Solibacillus isronensis* | BB | *+* | + | + | + | - | - | - |
| Endo47 | *Pantoea dispersa* | BB | *+* | - | + | + | + | - | + |
| Endo48 | *Lysinibacillus capsici* | BB | *+* | + | + | - | - | - | - |
| Endo49 | *Lysinibacillus odysseyi* | WYB | *+* | - | + | - | - | - | - |
| Endo50 | *Pseudomonas mosselii* | WEB | *+* | + | + | - | + | + | - |
| Endo51 | *Kosakonia cowanii* | BO | *+* | + | + | - | - | + | - |
| Endo54 | *Exiguobacterium indicum* | AB | *+* | - | - | - | - | + | - |
| Endo55 | *Pseudomonas mosselii* | BB | *+* | + | + | - | - | + | - |
| Endo56 | *Pseudomonas benzopyrenica* | BB | *+* | + | + | + | + | + | - |
| Endo57 | *Peribacillus castrilensis* | WYB | *+* | - | - | - | - | + | - |
| Endo58 | *Lysinibacillus odysseyi* | BB | *+* | + | + | - | - | - | - |
| Endo59 | *Pseudomonas siliginis* | AB | *+* | + | + | + | - | + | + |
| Endo60 | *Pseudomonas siliginis* | BB | *+* | - | + | - | - | + | - |
| Endo61 | *Pseudomonas simiae* | WYB | *+* | - | + | + | - | + | - |
| Endo62 | *Pseudomonas iranensis* | AB | *+* | - | + | + | - | + | + |
| Endo63 | *Solibacillus isronensis* | WYB | *+* | - | + | + | - | + | - |
| Endo64 | *Solibacillus isronensis* | BB | *+* | - | - | + | - | + | - |
| Endo65 | *Bacillus paralicheniformis* | WEB | *+* | + | - | + | - | + | + |
| Endo66 | *Bacillus anthracis* | WYB | *+* | - | + | * | - | + | - |
| Endo67 | *Bacillus paramycoides* | WYB | *+* | - | + | * | - | + | - |

**Table S4:** Combined results of plant species, biochemical traits and taxonomical of isolated rhizosphere strains. The values represent the mean ± standard deviation of three replicates. (+) indication of no production; (-) indication of no production; (*) indication of indefinite.

| Isolates | Taxonomic | Plant  species | Ammonia  production mg/mL | EPS Sucrose  mg/mL | EPS Glucose mg/mL | Biofilm production OD_600_ | Biofilm  10% PEG OD_600_ | IAA not L-trp  μg/ml | IAA L-trp  μg/ml |
| --- | --- | --- | --- | --- | --- | --- | --- | --- | --- |
| Rhizo1 | *Bacillus safensis* | WEB | 13,18 ± 0,06 | 36,32 ± 0,04 | 12,25 ± 0,01 | 0,08 ± 0,04 | 0,36 ± 0,41 | 24,50 ± 0,01 | 47,83 ± 0,00 |
| Rhizo2 | *Bacillus paramycoides* | BO | 25,85 ± 0,19 | 22,25 ± 0,01 | 22,69 ± 0,02 | - | 0,11 ± 0,00 | 12,80 ± 0,01 | 28,67 ± 0,00 |
| Rhizo3 | *Serratia odorifera* | BO | 16,02 ± 0,03 | 45,65 ± 0,07 | 34,42 ± 0,03 | - | - | 14,90 ± 0,01 | 23,25 ± 0,01 |
| Rhizo5 | *Piscinibacter defluvii* | AB | 24,73 ± 0,38 | 29,25 ± 0,06 | 18,64 ± 0,01 | - | 0,44 ± 0,07 | 86,60 ± 0,03 | 139,08 ± 0,01 |
| Rhizo7 | *Proteus cibi* | BO | 13,52 ± 0,02 | 21,92 ± 0,02 | 19,64 ± 0,02 | 0,04 ± 0,01 | 0,11 ± 0,02 | 101,20 ± 0,01 | 141,17 ± 0,01 |
| Rhizo8 | *Delftia sp.* | BO | 9,57 ± 0,11 | 33,25 ± 0,03 | 28,58 ± 0,01 | 0,04 ± 0,01 | 0,02 ± 0,00 | 29,50 ± 0,01 | 43,67 ± 0,01 |
| Rhizo9 | *Serratia odorifera* | WYB | 19,55 ± 0,22 | 44,52 ± 0,02 | 38,64 ± 0,04 | 0,29 ± 0,03 | 0,69 ± 0,13 | 37,00 ± 0,01 | 43,25 ± 0,00 |
| Rhizo10 | *Bacillus subtilis* | WEB | 12,13 ± 0,36 | 88,19 ± 0,03 | 35,25 ± 0,01 | 0,05 ± 0,00 | 0,20 ± 0,08 | 244,10 ± 0,03 | 197,42 ± 0,10 |
| Rhizo11 | *Bacillus paramycoides* | AB | 24,39 ± 0,49 | 28,45 ± 0,06 | 17,19 ± 0,02 | - | 0,01 ± 0,00 | 28,30 ± 0,00 | 101,17 ± 0,07 |
| Rhizo12 | *Morganella psychrotolerans* | WYB | 15,16 ± 0,05 | 36,45 ± 0,08 | 19,08 ± 0,03 | 0,27 ± 0,34 | 0,39 ± 0,09 | 138,30 ± 0,01 | 284,5 ± 0,01 |
| Rhizo13 | *Proteus cibi* | BB | 17,47 ± 0,07 | 25,85 ± 0,05 | 14,88 ± 0,02 | 0,09 ± 0,01 | 0,34 ± 0,03 | 87,00 ± 0,02 | 91,17 ± 0,00 |
| Rhizo14 | *Morganella psychrotolerans* | WYB | 16,04 ± 0,20 | 38,79 ± 0,06 | 20,25 ± 0,01 | 0,09 ± 0,01 | 0,15 ± 0,05 | 53,70 ± 0,01 | 114,5 ± 0,01 |
| Rhizo15 | *Morganella psychrotolerans* | WEB | 13,50 ± 0,26 | 26,79 ± 0,01 | 35,86 ± 0,02 | 0,19 ± 0,07 | 0,06 ± 0,01 | 105,30 ± 0,00 | 68,25 ± 0,01 |
| Rhizo16 | *Micrococcus luteus* | WEB | 17,81 ± 0,08 | 21,32 ± 0,00 | 25,36 ± 0,02 | - | - | 62,00 ± 0,02 | 82,42 ± 0,00 |
| Rhizo17 | *Microbacterium binotii* | WEB | 25,72 ± 0,02 | 36,12 ± 0,02 | 12,34 ± 0,01 | - | - | 64,10 ± 0,01 | 141,58 ± 0,03 |
| Rhizo18 | *Pantoea agglomerans* | WEB | 18,46 ± 0,77 | 34,25 ± 0,02 | 14,79 ± 0,01 | - | - | 42,00 ± 0,01 | 61,17 ± 0,01 |
| Rhizo19 | *Proteus mirabilis* | BO | 24,62 ± 0,62 | 23,65 ± 0,00 | 15,01 ± 0,01 | 0,38 ± 0,04 | 0,24 ± 0,02 | 145,80 ± 0,01 | 89,08 ± 0,01 |
| Rhizo20 | *Chryseobacterium cucumeris* | BB | 25,12 ± 0,88 | 18,85 ± 0,01 | 25,47 ± 0,03 | 0,16 ± 0,21 | 0,01 ± 0,01 | 8,80 ± 0,00 | 9,5 ± 0,00 |
| Rhizo21 | *Pseudomonas argentinensis* | BO | 20,88 ± 0,81 | 29,12 ± 0,01 | 26,25 ± 0,02 | 0,01 ± 0,01 | 0,02 ± 0,01 | 40,30 ± 0,00 | 74,5 ± 0,00 |
| Rhizo22 | *Chryseobacterium cucumeris* | BB | - | 46,52 ± 0,06 | 32,97 ± 0,02 | - | - | 31,20 ± 0,04 | 55,30 ± 0,04 |
| Rhizo23 | *Micrococcus yunnanensis* | BB | - | 26,45 ± 0,00 | 27,53 ± 0,02 | - | - | 57,80 ± 0,02 | 60,33 ± 0,01 |
| Rhizo24 | *Rhodococcus qingshengii* | WEB | - | 31,19 ± 0,01 | 31,19 ± 0,02 | 0,07 ± 0,01 | 0,08 ± 0,04 | 0,10 ± 0,00 | 27,83 ± 0,00 |
| Rhizo25 | *Pantoea agglomerans* | BB | - | - | - | - | - | - | - |
| Rhizo26 | *Bacillus safensis* | BB | - | - | - | - | - | - | - |
| Rhizo27 | *Rhodococcus qingshengii* | BB | - | - | - | - | - | - | - |
|  |  |  |  |  |  |  |  |  |  |
| Isolates | Taxonomic | Intercropping | Nitrogen fixation | EPS glucose | EPS sucrose | P solubilization | K solubilization | Siderophore | Cellulose |
| Rhizo1 | *Bacillus safensis* | WEB | + | + | + | + | - | + | - |
| Rhizo2 | *Bacillus paramycoides* | BO | + | + | + | - | - | - | - |
| Rhizo3 | *Serratia odorifera* | BO | + | + | + | + | + | + | - |
| Rhizo5 | *Piscinibacter defluvii* | AB | + | + | + | - | + | + | - |
| Rhizo7 | *Proteus cibi* | BO | + | + | + | - | - | + | - |
| Rhizo8 | *Delftia sp.* | BO | + | + | + | - | - | - | - |
| Rhizo9 | *Serratia odorifera* | WYB | + | + | + | + | + | + | + |
| Rhizo10 | *Bacillus subtilis* | WEB | + | + | + | - | - | + | - |
| Rhizo11 | *Bacillus paramycoides* | AB | + | + | + | + | - | - | - |
| Rhizo12 | *Morganella psychrotolerans* | WYB | + | + | + | - | - | + | - |
| Rhizo13 | *Proteus cibi* | BB | + | + | + | - | - | - | - |
| Rhizo14 | *Morganella psychrotolerans* | WYB | + | + | + | - | - | + | - |
| Rhizo15 | *Morganella psychrotolerans* | WEB | + | + | + | + | - | + | + |
| Rhizo16 | *Micrococcus luteus* | WEB | + | + | + | * | - | - | - |
| Rhizo17 | *Microbacterium binotii* | WEB | + | + | + | * | - | - | + |
| Rhizo18 | *Pantoea agglomerans* | WEB | + | + | + | * | - | + | + |
| Rhizo19 | *Proteus mirabilis* | BO | + | + | + | - | - | + | - |
| Rhizo20 | *Chryseobacterium cucumeris* | BB | + | + | + | - | - | + | - |
| Rhizo21 | *Pseudomonas argentinensis* | BO | + | + | + | - | - | + | - |
| Rhizo22 | *Chryseobacterium cucumeris* | BB | + | + | + | * | - | + | - |
| Rhizo23 | *Micrococcus yunnanensis* | BB | + | + | + | * | - | + | - |
| Rhizo24 | *Rhodococcus qingshengii* | WEB | + | + | + | - | - | + | - |
| Rhizo25 | *Pantoea agglomerans* | BB | + | + | + | * | - | * | + |
| Rhizo26 | *Bacillus safensis* | BB | + | + | + | * | * | * | - |
| Rhizo27 | *Rhodococcus qingshengii* | BB | + | + | + | * | * | * | * |

**Table S5:** The table presents the mean ± standard deviation (SD) of endophytic isolates growth responses for bacterial strains under drought, heat and salinity stress conditions. Growth was measured using optical density (OD_600_) after incubation, with higher OD values indicating greater tolerance to the stress conditions. The values represent the mean ± standard deviation of three independent replicates. (-) indicated no growth.

| Isolated | Taxonomic | Intercropping | Drought stress  Without PEG | Drought stress  10% PEG | Heat stress  40 °C | Heat stress  45 °C | Heat stress  55 °C | Saline stress 5%NaCl | Saline stress 10% NaCl |
| --- | --- | --- | --- | --- | --- | --- | --- | --- | --- |
| Endo1 | *Solibacillus silvestris* | BB | 0,35 ± 0,10 | 0,09 ± 0,04 | 0,13 ± 0,00 | 0,30 ± 0,01 | 0,10 ± 0,00 | 0,42 ± 0,01 | 0,06 ± 0,01 |
| Endo2 | *Kosakonia cowanii* | BO | 0,55 ± 0,01 | 0,23 ± 0,17 | 0,14 ± 0,02 | 0,10 ± 0,00 | 0,10 ± 0,00 | 0,59 ± 0,01 | 0,27 ± 0,01 |
| Endo3 | *Pseudomonas mosselii* | WYB | 0,54 ± 0,01 | 0,31 ± 0,02 | 0,32 ± 0,00 | 0,12 ± 0,00 | 0,15 ± 0,00 | 0,36 ± 0,00 | 0,06 ± 0,05 |
| Endo4 | *Solibacillus isronensis* | WYB | 0,53 ± 0,04 | 0,12 ± 0,05 | 0,32 ± 0,01 | 0,12 ± 0,00 | 0,10 ± 0,00 | 0,62 ± 0,72 | - |
| Endo5 | *Pseudomonas argentinensis* | BO | 0,44 ± 0,11 | 0,19 ± 0,09 | 0,14 ± 0,00 | 0,28 ± 0,03 | 0,16 ± 0,01 | 0,44 ± 0,32 | 0,42 ± 0,20 |
| Endo6 | *Bacillus paranthracis* | AB | 0,71 ± 0,08 | 0,36 ± 0,00 | 0,14 ± 0,02 | 0,24 ± 0,00 | 0,10 ± 0,00 | 0,42 ± 0,02 | 0,21 ± 0,01 |
| Endo7 | *Pantoea agglomerans* | BO | 0,49 ± 0,02 | 0,26 ± 0,02 | 0,17 ± 0,00 | 0,15 ± 0,00 | 0,10 ± 0,00 | 0,65 ± 0,02 | 0,19 ± 0,01 |
| Endo8 | *Pseudomonas granadensis* | BB | 0,50 ± 0,04 | 0,36 ± 0,26 | 0,14 ± 0,00 | 0,40 ± 0,07 | 0,13 ± 0,00 | 0,37 ± 0,01 | 0,38 ± 0,28 |
| Endo9 | *Achromobacter deleyi* | WYB | 0,60 ± 0,03 | 0,27 ± 0,03 | - | - | - | 0,10± 0,01 | 0,18 ± 0,06 |
| Endo10 | *Bacillus paranthracis* | BO | 0,64 ± 0,02 | 0,36 ± 0,01 | 0,36 ± 0,01 | 0,21± 0,01 | 0,10 ± 0,01 | 0,37 ± 0,01 | 0,59 ± 0,23 |
| Endo11 | *Solibacillus isronensis* | AB | 0,67 ± 0,05 | 0,13 ± 0,00 | 0,27 ± 0,00 | 0,22 ± 0,01 | 0,11 ± 0,00 | 0,58 ± 0,24 | 0,08 ± 0,01 |
| Endo12 | *Pantoea agglomerans* | WEB | 0,52 ± 0,06 | 0,40 ± 0,41 | 0,25 ± 0,01 | 0,10 ± 0,00 | 0,10 ± 0,00 | 0,44 ± 0,02 | 0,16 ± 0,02 |
| Endo13 | *Moraxella osloensis* | BB | 0,63 ± 0,01 | 0,23 ± 0,07 | 0,22 ± 0,00 | 0,42 ± 0,10 | 0,25 ± 0,00 | 0,28 ± 0,01 | 0,01 ± 0,01 |
| Endo14 | *Achromobacter deleyi* | BO | 0,65 ± 0,02 | 0,39 ± 0,02 | 0,19 ± 0,00 | 0,17 ± 0,00 | 0,12 ± 0,01 | 0,10 ± 0,00 | 0,07 ± 0,09 |
| Endo15 | *Enterobacter kobei* | AB | 0,65 ± 0,05 | 0,44 ± 0,03 | 0,32 ± 0,00 | 0,12 ± 0,01 | 0,15 ± 0,00 | 0,75 ± 0,14 | - |
| Endo16 | *Peribacillus frigoritolerans* | AB | 0,61 ± 0,04 | 0,30 ± 0,34 | 0,20 ± 0,00 | 0,17 ± 0,05 | 0,15 ± 0,03 | 0,46 ± 0,01 | 0,27 ± 0,17 |
| Endo18 | *Bacillus paralicheniformis* | WEB | 0,47 ± 0,09 | 0,19 ± 0,01 | 0,43 ± 0,01 | 0,22 ± 0,01 | 0,15 ± 0,01 | 0,85 ± 0,21 | 0,13 ± 0,01 |
| Endo19 | *Bacillus paranthracis* | AB | 0,59 ± 0,03 | 0,34 ± 0,05 | 0,20 ± 0,00 | 0,12 ± 0,00 | 0,06 ± 0,01 | 0,38 ± 0,02 | 0,14 ± 0,11 |
| Endo20 | *Pseudomonas mosselii* | BB | 0,55 ± 0,03 | 0,41 ± 0,02 | 0,16 ± 0,00 | 0,10 ± 0,01 | 0,11 ± 0,01 | 0,41 ± 0,00 | 0,21 ± 0,01 |
| Endo22 | *Solibacillus isronensis* | BB | 0,62 ± 0,03 | 0,39 ± 0,31 | 0,27 ± 0,00 | 0,15 ± 0,01 | 0,10 ± 0,00 | 0,36 ± 0,06 | 0,23 ± 0,09 |
| Endo23 | *Kocuria sediminis* | BB | 0,65 ± 0,10 | 0,30 ± 0,06 | 0,32 ± 0,00 | 0,14 ± 0,00 | 0,10 ± 0,00 | 0,53 ± 0,01 | 0,03 ± 0,01 |
| Endo24 | *Pseudomonas shirazensis* | BB | 0,44 ± 0,03 | 0,29 ± 0,01 | - | - | - | 0,36 ± 0,01 | 0,18 ± 0,01 |
| Endo26 | *Enterobacter kobei* | AB | 0,47 ± 0,04 | 0,41 ± 0,01 | 0,39 ± 0,00 | 0,14 ± 0,02 | 0,10 ± 0,01 | 0,62 ± 0,09 | 0,33 ± 0,20 |
| Endo27 | *Pseudoroseomonas rhizosphaerae* | BO | 0,57 ± 0,07 | 0,27 ± 0,14 | - | - | - | 0,26 ± 0,02 | 0,32 ± 0,03 |
| Endo28 | *Peribacillus castrilensis* | AB | 0,35 ± 0,11 | 0,18 ± 0,00 | 0,37 ± 0,01 | 0,21 ± 0,13 | 0,13 ± 0,07 | 0,59 ± 0,02 | 0,30 ± 0,20 |
| Endo29 | *Pseudomonas granadensis* | BB | 0,09 ± 0,10 | 0,22 ± 0,01 | - | - | - | 0,64 ± 0,01 | 0,12 ± 0,01 |
| Endo30 | *Lysinibacillus odysseyi* | BO | 0,37 ± 0,05 | 0,32 ± 0,01 | 0,21 ± 0,00 | 0,29 ± 0,00 | 0,12 ± 0,00 | 0,22 ± 0,14 | 0,16 ± 0,14 |
| Endo32 | *Pseudomonas mendocina* | BB | 0,33 ± 0,14 | 0,08 ± 0,02 | 0,19 ± 0,00 | 0,11 ± 0,00 | 0,10 ± 0,00 | 0,15 ± 0,01 | 0,37 ± 0,01 |
| Endo33 | *Pseudomonas simiae* | WEB | - | 0,11 ± 0,01 | - | - | - | 0,64 ± 0,01 | 0,25 ± 0,05 |
| Endo35 | *Staphylococcus succinus* | BB | - | 0,12 ± 0,01 | 0,34 ± 0,00 | 0,33 ± 0,00 | 0,29 ± 0,00 | 0,62 ± 0,13 | 0,24 ± 0,05 |
| Endo38 | *Bacillus paramycoides* | BO | 0,60 ± 0,06 | 0,52 ± 0,15 | 0,18 ± 0,00 | 0,30 ± 0,21 | 0,10 ± 0,00 | 0,80 ± 0,01 | 0,03 ± 0,03 |
| Endo39 | *Pseudomonas mosselii* | WYB | 0,48 ± 0,02 | 0,15 ± 0,02 | - | 0,13 ± 0,00 | 0,10 ± 0,00 | 0,56 ± 0,10 | 0,07 ± 0,03 |
| Endo40 | *Flavobacterium hibisci* | AB | 0,55 ± 0,03 | 0,04 ± 0,03 | 0,13 ± 0,00 | 0,20 ± 0,00 | 0,13 ± 0,00 | 0,33 ± 0,16 | 0,17 ± 0,11 |
| Endo42 | *Lysinibacillus odysseyi* | WYB | 0,64 ± 0,02 | 0,22 ± 0,06 | 0,40 ± 0,20 | 0,18 ± 0,00 | 0,14 ± 0,00 | 0,48 ± 0,01 | 0,12 ± 0,01 |
| Endo44 | *Pseudomonas mosselii* | AB | 0,55 ± 0,08 | 0,26 ± 0,04 | 0,13 ± 0,00 | 0,1 ± 0,00 | 0,10 ± 0,00 | 0,22 ± 0,01 | 0,02 ± 0,01 |
| Endo45 | *Solibacillus isronensis* | BB | 0,68 ± 0,04 | 0,06 ± 0,04 | 0,21 ± 0,00 | 0,18 ± 0,01 | 0,10 ± 0,01 | 0,27 ± 0,02 | 0,02 ± 0,00 |
| Endo47 | *Pantoea dispersa* | BB | 0,25 ± 0,03 | 0,14 ± 0,01 | - | - | - | - | - |
| Endo48 | *Lysinibacillus capsici* | BB | 0,18 ± 0,04 | 0,12 ± 0,02 | 0,19 ± 0,00 | 0,33 ± 0,01 | 0,10 ± 0,00 | 0,24 ± 0,03 | 0,06 ± 0,02 |
| Endo49 | *Lysinibacillus odysseyi* | WYB | 0,30 ± 0,04 | 0,27 ± 0,01 | 0,26 ± 0,01 | 0,27 ± 0,01 | 0,10 ± 0,00 | 0,68 ± 0,03 | 0,17 ± 0,03 |
| Endo50 | *Pseudomonas mosselii* | WEB | 0,55 ± 0,06 | 0,35 ± 0,01 | - | - | - | 0,41± 0,01 | 0,34 ± 0,00 |
| Endo51 | *Kosakonia cowanii* | BO | 0,31 ± 0,04 | 0,42 ± 0,08 | 0,16 ± 0,00 | 0,11 ± 0,00 | 0,10 ± 0,00 | 0,58± 0,01 | - |
| Endo54 | *Exiguobacterium indicum* | AB | 0,50 ± 0,06 | 0,43 ± 0,01 | 0,19 ± 0,00 | 0,27 ± 0,00 | 0,15 ± 0,00 | 0,49 ± 0,01 | 0,24 ± 0,03 |
| Endo55 | *Pseudomonas mosselii* | BB | 0,53 ± 0,02 | 0,36 ± 0,00 | 0,26 ± 0,00 | 0,1 ± 0,00 | 0,10 ± 0,00 | 0,70 ± 0,01 | 0,31 ± 0,02 |
| Endo56 | *Pseudomonas benzopyrenica* | BB | 0,45 ± 0,09 | 0,31 ± 0,16 | - | - | - | 0,27 ± 0,00 | 0,17 ± 0,01 |
| Endo57 | *Peribacillus castrilensis* | WYB | 0,70 ± 0,05 | 0,35 ± 0,00 | 0,22 ± 0,00 | 0,21 ± 0,00 | 0,13 ± 0,00 | 0,30 ± 0,01 | 0,55 ± 0,12 |
| Endo58 | *Lysinibacillus odysseyi* | BB | 0,71 ± 0,01 | 0,31 ± 0,01 | 0,18 ± 0,00 | 0,24 ± 0,00 | 0,10 ± 0,00 | 0,48 ± 0,04 | 0,23 ± 0,03 |
| Endo59 | *Pseudomonas siliginis* | AB | 0,69 ± 0,08 | 0,32 ± 0,01 | 0,35 ± 0,01 | 0,51 ± 0,01 | 0,13 ± 0,00 | 0,46 ± 0,00 | 0,34 ± 0,01 |
| Endo60 | *Pseudomonas siliginis* | BB | 0,40 ± 0,14 | 0,18 ± 0,11 | 0,19 ± 0,00 | 0,14 ± 0,00 | 0,10 ± 0,01 | 0,29 ± 0,05 | - |
| Endo61 | *Pseudomonas simiae* | WYB | 0,69 ± 0,04 | 0,36 ± 0,02 | - | - | - | 0,31 ± 0,00 | 0,41 ± 0,05 |
| Endo62 | *Pseudomonas iranensis* | AB | 0,65 ± 0,03 | 0,32 ± 0,01 | 0,17 ± 0,00 | 0,13 ± 0,00 | 0,12 ± 0,01 | 0,15 ± 0,00 | 0,02 ± 0,01 |
| Endo63 | *Solibacillus isronensis* | WYB | 0,55 ± 0,13 | 0,35 ± 0,03 | 0,26 ± 0,01 | 0,20 ± 0,00 | 0,10 ± 0,00 | 0,25 ± 0,02 | 0,09 ± 0,00 |
| Endo64 | *Solibacillus isronensis* | BB | 0,24 ± 0,07 | 0,18 ± 0,06 | 0,23 ± 0,00 | 0,18 ± 0,00 | 0,10 ± 0,01 | 0,19 ± 0,14 | 0,03 ± 0,01 |
| Endo65 | *Bacillus paralicheniformis* | WEB | 0,69 ± 0,06 | 0,32 ± 0,00 | 0,17 ± 0,00 | 0,15 ± 0,03 | 0,13 ± 0,01 | 0,26 ± 0,12 | 0,07 ± 0,00 |
| Endo66 | *Bacillus anthracis* | WYB | 0,66 ± 0,02 | 0,43 ± 0,26 | 0,18 ± 0,00 | 0,19 ± 0,00 | 0,10 ± 0,00 | 0,24 ± 0,03 | 0,04 ± 0,03 |
| Endo67 | *Bacillus paramycoides* | WYB | 0,63 ± 0,02 | 0,06 ± 0,03 | 0,14 ± 0,00 | 0,32 ± 0,00 | 0,10 ± 0,00 | 0,24 ± 0,00 | 0,12 ± 0,03 |

**Table S6:** The table presents the mean ± standard deviation (SD) of rhizosphere isolates growth responses for bacterial strains under drought, heat and salinity stress conditions. Growth was measured using optical density (OD_600_) after incubation, with higher OD values indicating greater tolerance to the stress conditions. The values represent the mean ± standard deviation of three independent replicates. (-) indicated no growth.

| Isolated | Taxonomic | Intercropping | Drought stress  Without PEG | Drought stress  10% PEG | Heat stress  40 °C | Heat stress  45 °C | Heat stress  55 °C | Saline stress 5%NaCl | Saline stress 10% NaCl |
| --- | --- | --- | --- | --- | --- | --- | --- | --- | --- |
| Rhizo1 | *Bacillus safensis* | WEB | 1,21 ± 0,01 | 0,69 ± 0,01 | 0,26 ± 0,05 | 0,30 ± 0,00 | 0,02 ± 0,00 | 0,18 ± 0,00 | 0,15 ± 0,00 |
| Rhizo2 | *Bacillus paramycoides* | BO | 0,95 ± 0,00 | 0,65 ± 0,00 | 0,26 ± 0,02 | 0,23 ± 0,01 | 0,02 ± 0,00 | 0,24 ± 0,00 | 0,05 ± 0,00 |
| Rhizo3 | *Serratia odorifera* | BO | 0,77 ± 0,00 | 0,38 ± 0,03 | - | - | - | 0,52 ± 0,00 | 0,04 ± 0,00 |
| Rhizo5 | *Piscinibacter defluvii* | AB | 0,83 ± 0,01 | 0,59 ± 0,01 | 0,24 ± 0,02 | 0,19 ± 0,01 | 0,02 ± 0,00 | 0,52 ± 0,00 | 0,06 ± 0,00 |
| Rhizo7 | *Proteus cibi* | BO | 0,78 ± 0,00 | 0,69 ± 0,00 | 0,22 ± 0,01 | 0,14 ± 0,01 | 0,02 ± 0,00 | 0,51 ± 0,00 | 0,07 ± 0,00 |
| Rhizo8 | *Delftia sp.* | BO | 0,57 ± 0,00 | 0,34 ± 0,01 | - | - | - | 0,03 ± 0,00 | 0,04 ± 0,00 |
| Rhizo9 | *Serratia odorifera* | WYB | 0,63 ± 0,01 | 0,51 ± 0,01 | - | - | - | 0,54 ± 0,00 | 0,07 ± 0,00 |
| Rhizo10 | *Bacillus subtilis* | WEB | 1,06 ± 0,00 | 0,80 ± 0,01 | 0,25 ± 0,06 | 0,33 ± 0,02 | 0,03 ± 0,00 | 0,17 ± 0,00 | 0,07 ± 0,00 |
| Rhizo11 | *Bacillus paramycoides* | AB | 0,80 ± 0,01 | 0,77 ± 0,00 | 0,21 ± 0,02 | 0,27 ± 0,02 | 0,02 ± 0,00 | 0,14 ± 0,00 | 0,05 ± 0,00 |
| Rhizo12 | *Morganella psychrotolerans* | WYB | 0,80 ± 0,01 | 0,30 ± 0,02 | - | - | - | 0,49 ± 0,00 | 0,05 ± 0,00 |
| Rhizo13 | *Proteus cibi* | BB | 0,88 ± 0,01 | 0,46 ± 0,01 | - | - | - | 0,02 ± 0,00 | 0,05 ± 0,00 |
| Rhizo14 | *Morganella psychrotolerans* | WYB | 0,98 ± 0,01 | 0,37 ± 0,06 | 0,18 ± 0,08 | 0,26 ± 0,03 | 0,03 ± 0,01 | 0,53 ± 0,00 | 0,12 ± 0,00 |
| Rhizo15 | *Morganella psychrotolerans* | WEB | 0,73 ± 0,01 | 0,38 ± 0,00 | 0,18 ± 0,12 | 0,30 ± 0,01 | 0,04 ± 0,01 | 0,44 ± 0,00 | 0,12 ± 0,00 |
| Rhizo16 | *Micrococcus luteus* | WEB | - | - | - | - | - | 0,28 ± 0,00 | 0,15 ± 0,00 |
| Rhizo17 | *Microbacterium binotii* | WEB | - | - | - | - | - | 0,54 ± 0,00 | 0,05 ± 0,00 |
| Rhizo18 | *Pantoea agglomerans* | WEB | - | - | - | - | - | 0,23 ± 0,00 | 0,04 ± 0,00 |
| Rhizo19 | *Proteus mirabilis* | BO | 0,86 ± 0,00 | 0,76 ± 0,00 | - | - | - | 0,63 ± 0,00 | 0,16 ± 0,00 |
| Rhizo20 | *Chryseobacterium cucumeris* | BB | 0,79 ± 0,01 | 0,51 ± 0,06 | - | - | - | - | - |
| Rhizo21 | *Pseudomonas argentinensis* | BO | 0,39 ± 0,00 | 0,45 ± 0,00 | - | - | - | - | - |
| Rhizo22 | *Chryseobacterium cucumeris* | BB | - | - | 0,11 ± 0,02 | - | 0,01 ± 0,00 | 0,32 ± 0,00 | 0,04 ± 0,00 |
| Rhizo23 | *Micrococcus yunnanensis* | BB | - | - | 0,24 ± 0,01 | 0,25 ± 0,06 | 0,02 ± 0,00 | 0,23 ± 0,00 | 0,17 ± 0,00 |
| Rhizo24 | *Rhodococcus qingshengii* | WEB | 0,55 ± 0,01 | 0,36 ± 0,00 | 0,22 ± 0,01 | 0,24 ± 0,03 | 0,04 ± 0,00 | 0,33 ± 0,00 | 0,13 ± 0,00 |
| Rhizo25 | *Pantoea agglomerans* | BB | - | - | - | - | - | - | - |
| Rhizo26 | *Bacillus safensis* | BB | - | - | 0,20 ± 0,01 | 0,24 ± 0,11 | 0,01 ± 0,00 | 0,17 ± 0,00 | 0,10 ± 0,00 |
| Rhizo27 | *Rhodococcus qingshengii* | BB | - | - | 0,24 ± 0,02 | 0,19 ± 0,08 | 0,02 ± 0,01 | 0,47 ± 0,00 | 0,04 ± 0,00 |

**Table S7:** Enzyme activity of endophytic strains isolated from intercropping plants. (-) indicated no enzyme production, (+) represented moderate enzyme activity, and (++) demonstrated high enzyme activity.

| Isolated | Taxonomy | ACC deaminase | Lipase | Xylanase | Amylase | Cellulase | Protease |
| --- | --- | --- | --- | --- | --- | --- | --- |
| Endo1 | *Solibacillus silvestris* | - | + | + | ++ | + | ++ |
| Endo2 | *Kosakonia cowanii* | + | ++ | + | + | - | - |
| Endo3 | *Pseudomonas mosselii* | - | ++ | - | ++ | - | - |
| Endo4 | *Solibacillus isronensis* | - | + | + | + | + | + |
| Endo5 | *Pseudomonas argentinensis* | - | ++ | + | + | - | - |
| Endo6 | *Bacillus paranthracis* | - | - | - | - | ++ | + |
| Endo7 | *Pantoea agglomerans* | + | ++ | + | ++ | + | - |
| Endo8 | *Pseudomonas granadensis* | - | + | + | + | + | + |
| Endo9 | repeat 16s | + | - | - | - | - | - |
| Endo10 | *Bacillus paranthracis* | - | + | + | ++ | + | + |
| Endo11 | *Solibacillus isronensis* | + | + | ++ | + | + | ++ |
| Endo12 | *Pantoea agglomerans* | + | + | - | - | - | - |
| Endo13 | *Moraxella osloensis* | - | + | + | - | + | + |
| Endo14 | *Achromobacter deleyi* | + | ++ | + | - | - | ++ |
| Endo15 | *Enterobacter kobei* | + | - | + | - | - | - |
| Endo16 | *Peribacillus frigoritolerans* | + | - | + | ++ | + | ++ |
| Endo18 | *Bacillus paralicheniformis* | + | + | - | - | - | + |
| Endo19 | *Bacillus paranthracis* | - | - | - | - | - | - |
| Endo20 | *Pseudomonas mosselii* | + | - | - | - | - | - |
| Endo22 | *Solibacillus isronensis* | + | + | ++ | + | ++ | ++ |
| Endo23 | *Kocuria sediminis* | + | - | - | - | - | - |
| Endo24 | *Pseudomonas shirazensis* | + | - | + | ++ | - | - |
| Endo26 | *Enterobacter kobei* | + | - | ++ | - | - | - |
| Endo27 | *Pseudoroseomonas rhizosphaerae* | + | + | - | - | ++ | ++ |
| Endo28 | *Peribacillus castrilensis* | + | - | + | + | - | + |
| Endo29 | *Pseudomonas granadensis* | - | - | - | - | - | + |
| Endo30 | *Lysinibacillus odysseyi* | + | ++ | ++ | + | + | ++ |
| Endo32 | *Pseudomonas mendocina* | - | + | ++ | ++ | + | ++ |
| Endo33 | *Pseudomonas simiae* | + | + | - | - | - | + |
| Endo35 | *Staphylococcus succinus* | + | + | + | ++ | - | - |
| Endo38 | *Bacillus paramycoides* | + | + | ++ | + | + | + |
| Endo39 | *Pseudomonas mosselii* | + | ++ | + | ++ | + | ++ |
| Endo40 | *Flavobacterium hibisci* | - | + | ++ | + | ++ | + |
| Endo42 | *Lysinibacillus odysseyi* | - | ++ | + | + | + | ++ |
| Endo44 | *Pseudomonas mosselii* | - | + | + | + | - | ++ |
| Endo45 | *Solibacillus isronensis* | + | + | ++ | + | - | + |
| Endo47 | *Pantoea dispersa* | + | - | ++ | ++ | ++ | ++ |
| Endo48 | *Lysinibacillus capsici* | + | - | ++ | + | + | + |
| Endo49 | *Lysinibacillus odysseyi* | + | - | + | ++ | - | ++ |
| Endo50 | *Pseudomonas mosselii* | - | - | + | + | + | - |
| Endo51 | *Kosakonia cowanii* | + | + | + | ++ | - | - |
| Endo54 | *Exiguobacterium indicum* | + | ++ | + | ++ | + | ++ |
| Endo55 | *Pseudomonas mosselii* | + | ++ | + | ++ | - | + |
| Endo56 | *Pseudomonas benzopyrenica* | + | ++ | + | ++ | ++ | + |
| Endo57 | *Peribacillus castrilensis* | + | + | ++ | + | ++ | ++ |
| Endo58 | *Lysinibacillus odysseyi* | + | + | - | - | + | - |
| Endo59 | *Pseudomonas siliginis* | + | - | - | - | - | - |
| Endo60 | *Pseudomonas siliginis* | - | + | ++ | ++ | ++ | + |
| Endo61 | *Pseudomonas simiae* | + | + | ++ | + | ++ | ++ |
| Endo62 | *Pseudomonas iranensis* | - | ++ | + | ++ | ++ | + |
| Endo63 | *Solibacillus isronensis* | + | - | ++ | ++ | + | - |
| Endo64 | *Solibacillus isronensis* | - | ++ | ++ | + | ++ | + |
| Endo65 | *Bacillus paralicheniformis* | + | - | + | ++ | - | ++ |
| Endo66 | *Bacillus anthracis* | - | - | + | + | ++ | + |
| Endo67 | *Bacillus paramycoides* | - | + | + | ++ | - | ++ |

**Table S8:** Enzyme activity of rhizosphere strains isolated from intercropping plants. (-) indicated no enzyme production, (+) represented moderate enzyme activity, and (++) demonstrated high enzyme activity. (*) indication of indefinite.

| Isolated | Taxonomy | ACC deaminase | Lipase | Xylanase | Amylase | Cellulase | Protease |
| --- | --- | --- | --- | --- | --- | --- | --- |
| Rhizo1 | *Bacillus safensis* |  | ++ | ++ | ++ | ++ | - |
| Rhizo2 | *Bacillus paramycoides* |  | ++ | - | + | + | ++ |
| Rhizo3 | *Serratia odorifera* |  | - | ++ | - | + | ++ |
| Rhizo5 | *Piscinibacter defluvii* |  | ++ | + | - | ++ | ++ |
| Rhizo7 | *Proteus cibi* |  | - | - | + | ++ | ++ |
| Rhizo8 | *Delftia sp.* |  | + | + | ++ | - | + |
| Rhizo9 | *Serratia odorifera* |  | + | + | - | + | + |
| Rhizo10 | *Bacillus subtilis* |  | - | - | + | - | - |
| Rhizo11 | Bacillus paramycoides |  | ++ | ++ | ++ | ++ | ++ |
| Rhizo12 | *Morganella psychrotolerans* |  | - | + | - | - | - |
| Rhizo13 | *Proteus cibi* |  | + | ++ | + | + | ++ |
| Rhizo14 | *Morganella psychrotolerans* |  | ++ | + | ++ | ++ | + |
| Rhizo15 | *Morganella psychrotolerans* |  | - | + | - | - | + |
| Rhizo16 | *Micrococcus luteus* |  | ++ | + | - | + | + |
| Rhizo17 | *Microbacterium binotii* |  | * | * | * | * | * |
| Rhizo18 | *Pantoea agglomerans* |  | * | * | * | * | * |
| Rhizo19 | *Proteus mirabilis* |  | + | ++ | + | - | - |
| Rhizo20 | *Chryseobacterium cucumeris* |  | - | - | - | ++ | - |
| Rhizo21 | *Pseudomonas argentinensis* |  | ++ | + | + | ++ | ++ |
| Rhizo22 | *Chryseobacterium cucumeris* |  | + | - | ++ | + | ++ |
| Rhizo23 | *Micrococcus yunnanensis* |  | - | + | - | + | ++ |
| Rhizo24 | *Rhodococcus qingshengii* |  | ++ | - | + | - | - |
| Rhizo25 | *Pantoea agglomerans* |  | * | * | * | * | * |
| Rhizo26 | *Bacillus safensis* |  | - | ++ | - | - | - |
| Rhizo27 | *Rhodococcus qingshengii* |  | - | + | - | + | ++ |

Fig. S1: *Screening of PGPR traits in selected bacterial strains.* Phosphate and potassium solubilization indicated by a clean halo zone (a;b); Siderophore production determined by a blue halo zone around the colony (c); Cellulose production determined by a clear halo zone around the colony (d).


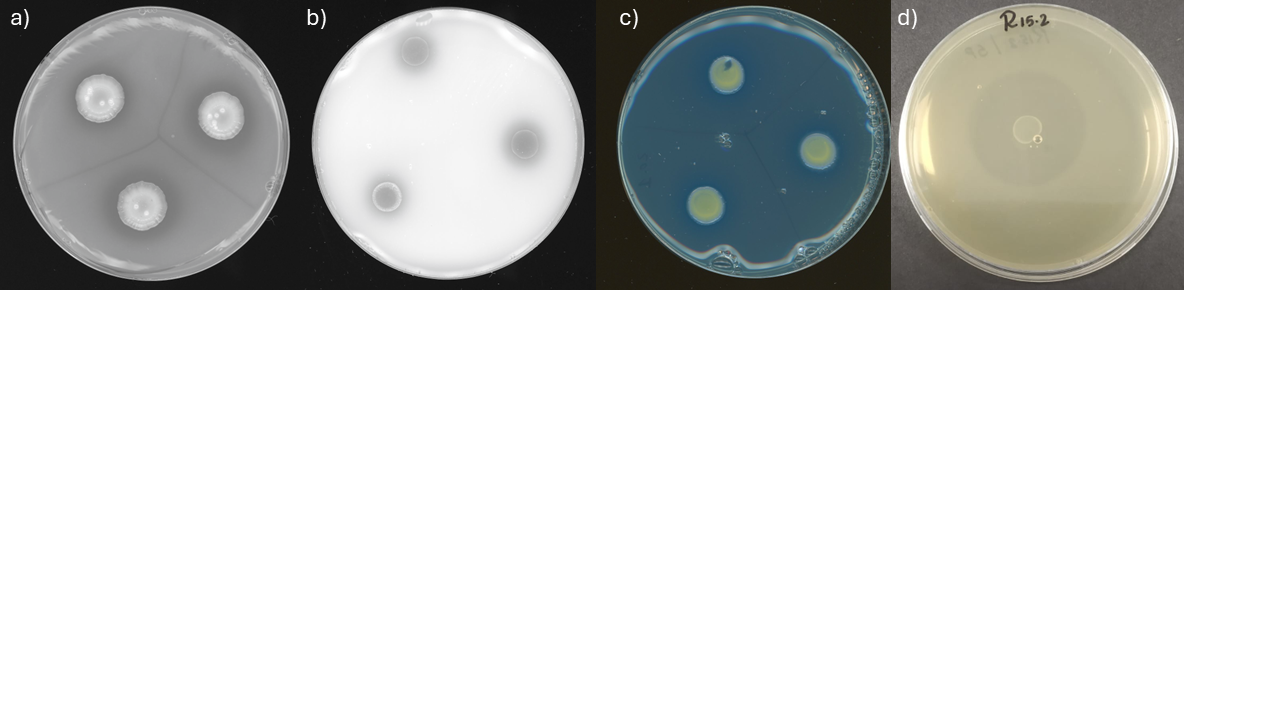


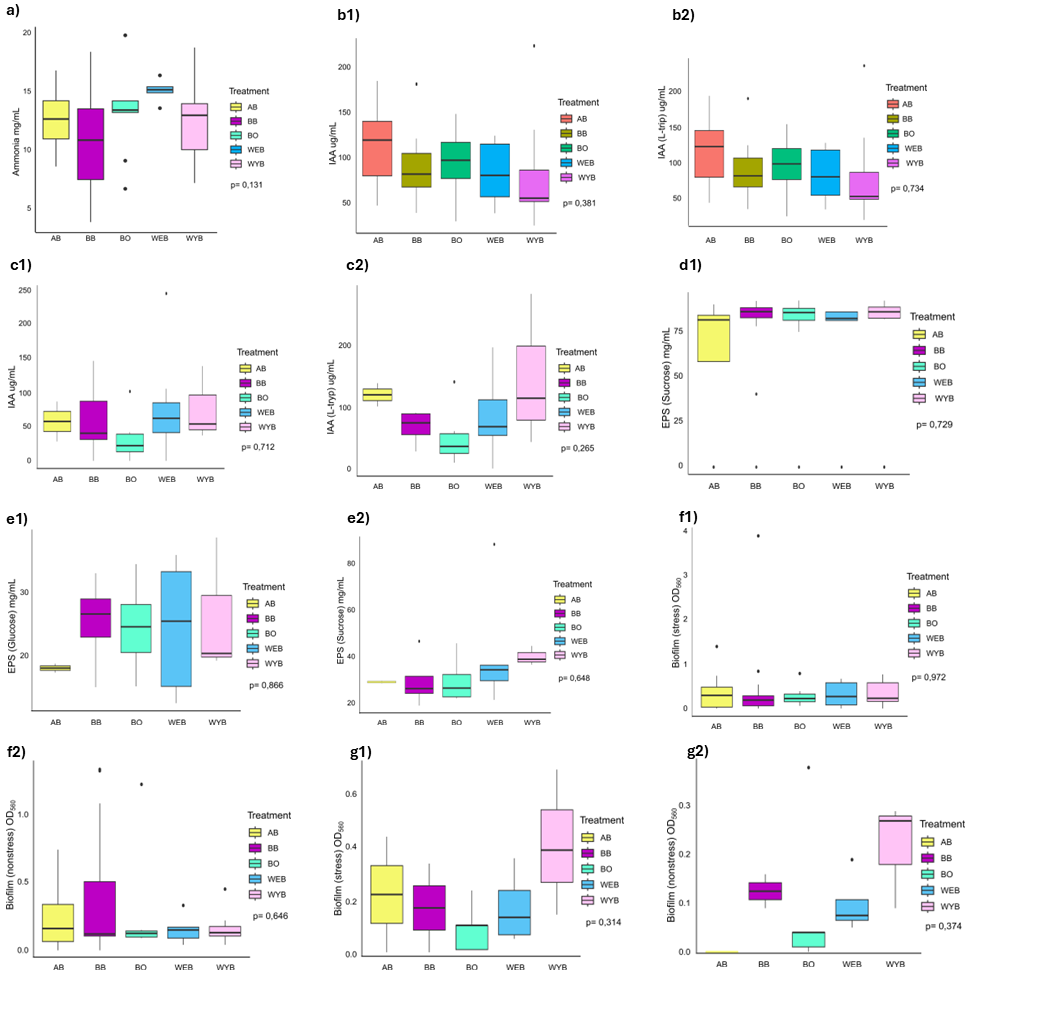


Fig. S2: Comparative analysis of endophyte and rhizosphere isolates between the intercropping and monocropping system. (a) Ammonia production by the endophyte isolates; (b1) IAA production by the endophyte isolates without L-trip; (b2) IAA production by endophyte isolates with L-trip; (c1) IAA production by the rhizosphere isolates without L-trip; (c2) IAA production by the rhizosphere isolates with L-trip isolates; (d1) EPS-forming by endophyte isolates using sucrose and glucose (d2); (e1) EPS-forming by rhizosphere isolates using sucrose and glucose (e2); (f1) Biofilm-forming by endophyte isolates under drought stress conditions and nonstress (f2); (g1) Biofilm-forming by rhizosphere isolates under drought stress conditions and nonstress (g2). Collor represents: yellow (AB - Alfalfa intercropping with Broad beans); purple (BB - Barley intercropping with Mustard); green (BO - Barley monocropping); blue (WEB - Egyptian wheat intercropping with Broad beans); pink (WYB - Wheat yokara intercropping with Broad beans). Data were analyzed using analysis of variance (ANOVA), bars represent mg/mL mean values from triplicate experiments, and error bars indicate standard deviations. Significant difference between treatments was evaluated at p < 0.05.
